# Dietary Restriction and Medical Therapy Drives PPARα-Regulated Improvements in Early Diabetic Kidney Disease in Male Rats

**DOI:** 10.1042/CS20220205

**Published:** 2022-11-11

**Authors:** William P. Martin, Meera Nair, Yeong H.D. Chuah, Daniel Malmodin, Anders Pedersen, Sanna Abrahamsson, Michaela Hutter, Mahmoud Abdelaal, Jessie A. Elliott, Naomi Fearon, Hans Eckhardt, Catherine Godson, Eoin P. Brennan, Lars Fändriks, Carel W. le Roux, Neil G. Docherty

**Affiliations:** 1 Diabetes Complications Research Centre, School of Medicine, Conway Institute of Biomolecular and Biomedical Research, University College Dublin, Belfield, D04 V1W8 Dublin, Ireland; 2 Swedish NMR Centre, University of Gothenburg, 40530 Gothenburg, Sweden; 3 Bioinformatics Core Facility, Sahlgrenska Academy, University of Gothenburg, 40530 Gothenburg, Sweden; 4 Institute of Clinical Sciences, Sahlgrenska Academy, University of Gothenburg, 40530 Gothenburg, Sweden; 5 Diabetes Research Group, Ulster University, Coleraine BT52 1SA, UK

**Keywords:** diabetic kidney disease, fatty acid oxidation, metabolome, mitochondria, obesity, peroxisome, PPARα, preclinical models, transcriptome, weight loss

## Abstract

The attenuation of diabetic kidney disease (DKD) by metabolic surgery is enhanced by pharmacotherapy promoting renal fatty acid oxidation (FAO). Using the Zucker Diabetic Fatty and Zucker Diabetic Sprague Dawley rat models of DKD, we conducted studies to determine if these effects could be replicated with a non-invasive bariatric mimetic intervention. Metabolic control and renal injury were compared in rats undergoing a dietary restriction plus medical therapy protocol (DMT; fenofibrate, liraglutide, metformin, ramipril, and rosuvastatin) and *ad libitum*-fed controls. The global renal cortical transcriptome and urinary ^1^H-NMR metabolomic profiles were also compared. Kidney cell type-specific and medication-specific transcriptomic responses were explored through *in silico* deconvolution. Transcriptomic and metabolomic correlates of improvements in kidney structure were defined using a molecular morphometric approach. The DMT protocol led to ~20% weight loss, normalised metabolic parameters and was associated with reductions in indices of glomerular and proximal tubular injury. The transcriptomic response to DMT was dominated by changes in fenofibrate- and PPARα-governed peroxisomal and mitochondrial FAO transcripts localizing to the proximal tubule. DMT induced urinary excretion of PPARα-regulated metabolites involved in nicotinamide metabolism and reversed DKD-associated changes in the urinary excretion of TCA cycle intermediates. FAO transcripts and urinary nicotinamide and TCA cycle metabolites were moderately-to-strongly correlated with improvements in glomerular and proximal tubular injury. Weight loss plus pharmacological PPARα agonism is a promising means of attenuating DKD.

## Introduction

Diabetic kidney disease (DKD) is the leading cause of end-stage kidney disease (ESKD) [4, 5]. Obesity is common in people with chronic kidney disease (CKD), with reported prevalence rates ranging from 35-44% [6, 7]. Despite being an independent risk factor for the onset and progression of DKD [8], intentional weight loss strategies are not yet routinely deployed in the management of this condition [6]. Emerging evidence does, however, support a possible role for metabolic surgery in the treatment of DKD [9, 10]. For example, in a randomised study of Roux-en-Y gastric bypass (RYGB) in patients with type 2 diabetes and microalbuminuria, remission of albuminuria at 24-month follow-up was greater following RYGB plus medications (82%) compared with medications alone (55%) [11].

In the Zucker Diabetic Fatty (ZDF) and Zucker Diabetic Sprague Dawley (ZDSD) rat models of DKD, we demonstrated that RYGB reduces glomerular injury in association with downregulation of fibrosis and inflammation pathways at the renal transcriptomic pathway level [12–16]. Whilst impaired proximal tubular fatty acid oxidation (FAO) is now recognised as a key driver of DKD-associated tubulointerstitial fibrosis and progressive renal functional decline [17, 18], we did not detect transcriptomic induction of FAO following RYGB-induced weight loss [14–16]. However, other authors have demonstrated increased renal expression of key regulators of FAO, including peroxisome proliferator-activated receptor-alpha (PPARα) and 5′ adenosine monophosphate-activated protein kinase (AMPK), following metabolic surgery in preclinical models of DKD [19, 20]. Accordingly, in the ZDSD rat model of DKD, we demonstrated that the renoprotective effects of RYGB could be enhanced when combined with FAO-directed pharmacotherapy for type 2 diabetes which strongly induced proximal tubular FAO [15].

Whilst this combination of metabolic surgery plus FAO-directed pharmacotherapy is a promising means of attenuating DKD progression [15], the invasive nature of surgically-induced weight loss may hamper its clinical translation. It may be difficult to convince patients with mild-moderate obesity that the reduction in renal and cardiovascular risk after metabolic surgery outweighs the risks of postoperative complications, which increase with advancing CKD stage [21], particularly for a disease such as DKD which is often asymptomatic until its advanced stages [22]. Aside from surgical complications, a non-invasive weight loss intervention could also be more feasibly scaled to larger numbers of eligible patients as it would obviate the need for an elective surgical procedure and associated wait times, which have increased for metabolic surgery over the past decade [23].

With recent advances in pharmacotherapy for obesity, the possibility of combining lifestyle intervention with medications to achieve sustained surgery-equivalent weight loss is becoming increasingly apparent. For example, randomisation to the glucagon-like peptide-1 receptor agonist (GLP1RA) semaglutide 2.4 mg once weekly plus lifestyle intervention resulted in 10-15% weight loss by follow-up at week 68 in patients with overweight or obesity, with or without type 2 diabetes [24, 25]. In an RCT of the dual GLP-1 and glucose-dependent insulinotropic polypeptide (GIP) receptor agonist tirzepatide (5, 10, and 15 mg once weekly) vs semaglutide 1 mg once weekly as add-on therapy to metformin in patients with type 2 diabetes, reductions in body weight at week 40 were greater at all 3 evaluated doses of tirzepatide compared with semaglutide [26]. Indeed, the 13.1% reduction in body weight observed with tirzepatide 15 mg once weekly was approximately double that achieved with semaglutide 1 mg once weekly (6.7%) over the same timeframe [26]. Furthermore, some of these medications have been demonstrated to have renoprotective effects in people with type 2 diabetes. For example, in a post-hoc analysis of the Semaglutide Unabated Sustainability in Treatment of Type 2 Diabetes (SUSTAIN) 1-7 RCTs, semaglutide was associated with marked reductions in albuminuria in people with type 2 diabetes in a manner similar to that reported following liraglutide [27, 28].

A non-invasive weight loss strategy may also avoid maladaptive consequences of metabolic surgery, which may have particularly deleterious consequences in people with DKD. For example, nutritional deficiencies in iron, calcium, and vitamin D after metabolic surgery may exacerbate anaemia and mineral bone disease as complications of DKD [29]. Given that anaemia is an important contributing factor to cardiovascular complications amongst patients with DKD [30], the avoidance of nutritional deficiencies may be important to optimising both cardiovascular and renal outcomes with intentional weight loss interventions in DKD. Enteric hyperoxaluria is also common following certain metabolic surgeries, such as RYGB [31]. While enteric hyperoxaluria is controllable with appropriate multi-disciplinary care [32], the avoidance of this complication with non-surgical approaches may have an overall benefit on net renal outcomes in people with DKD.

As such, in the present study, we assessed the impact of a dietary restriction plus medical therapy (DMT) intervention on renal injury endpoints, the renal cortical transcriptome, and the urinary metabolome in the ZDF and ZDSD rat models of DKD. The DMT intervention served as a bariatric mimetic control in previous preclinical studies of RYGB in our group [14, 15], and consisted of dietary restriction to RYGB-equivalent weight loss (~20% of baseline body weight) and treatment with fenofibrate, liraglutide, metformin, ramipril, and rosuvastatin. Aside from the impact on body weight, dietary restriction has been shown to activate PPARα-governed renal FAO to abrogate age-associated renal fibrosis [33]. All medications used in the DMT intervention are routinely deployed in the management of type 2 diabetes. Ramipril was included as renin-angiotensin-aldosterone system (RAAS) blockade is the backbone of DKD management [34]. Metformin, rosuvastatin and fenofibrate were included as, by convergent mechanisms, each drug can stimulate FAO [15, 35–37]. Liraglutide was administered to mimic the post-prandial increase in GLP-1 which is implicated in improvements in metabolic control and renal outcomes after metabolic surgery [38–41]. As we previously showed that RYGB-induced weight loss synergised with pharmacotherapy to reduce renal injury in association with activation of PPARα-mediated peroxisomal and mitochondrial FAO [15], we hypothesised that these beneficial effects could be replicated in a non-invasive fashion with DMT.

## Materials And Methods

### Animal studies

Experiments were undertaken at the University College Dublin Biomedical Facility under governmental project license (Health Products Regulatory Authority – AE18982/P084). Ethical approval for the animal study protocols was granted by the University College Dublin Animal Research Ethics Committee (approval reference AREC-15-32). Adult male ZDF (fa/fa) rats and lean control (fa/+, n=6) rats (Charles River Laboratories, France and UK) were acquired at 6-weeks old while adult male ZDSD rats and Sprague Dawley control (SD, n=6) rats (Crown BioScience) were acquired at 14-weeks old. All rats were provided with water and Purina 5008 rodent chow (Nestle Purina, St. Louis, MO). In both experiments, body weight and glycaemia-matched ZDF and ZDSD rats were allocated to either a sham disease control (SHAM, n=6 ZDF and n=7 ZDSD) or a dietary restriction plus medical therapy group (DMT, n=9 ZDF and n=6 ZDSD).

The DMT intervention consisted of dietary restriction to achieve 20% weight loss as well as the daily administration of multi-agent pharmacotherapy including 100 mg/kg fenofibrate (Mylan Pharma, Canonsburg, PA), 300 mg/kg metformin (Teva Pharma, Petah Tikva, Israel), 1 mg/kg ramipril (Sanofi, Paris, France), 10 mg/kg rosuvastatin (Teva Pharma), and 1 mg/kg (ZDF) or 0.2 mg/kg (ZDSD) liraglutide. Fenofibrate, metformin, ramipril, and rosuvastatin were incorporated into daily chow rations, while liraglutide was administered by subcutaneous injection. The dietary restriction protocol involved providing individually caged rats with a jar of Purina 5008 chow pre-soaked with a cocktail of the orally delivered medications. Rations were provided daily at 15:00 immediately after subcutaneous liraglutide administration and were started at 16g per rat per day. This equated to an approximately 50% reduction in food intake based on average *ad libitum* daily intake of hyperphagic ZDF and ZDSD rats. Ration quantity was maintained or titrated up and down daily in 2g increments or decrements as required to support the achievement and stable maintenance of approximately 20% weight loss within 1 month of starting the protocol.

**Figure 1:**
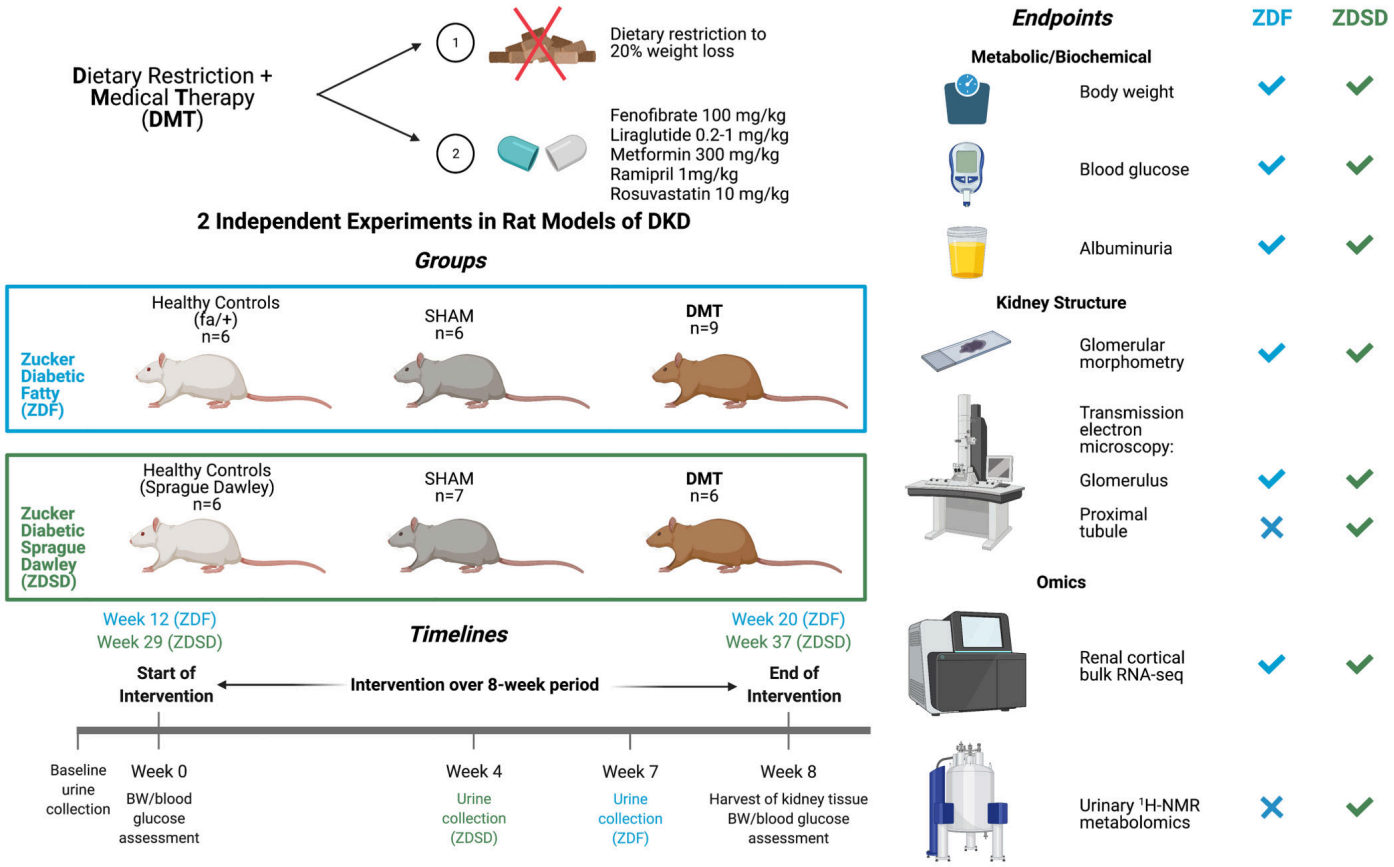
Multi-level characterisation of the impact of DMT on the evolution of DKD across two experimental models. Created with BioRender.com. ^1^H-NMR, proton nuclear magnetic resonance; BW, body weight; DKD, diabetic kidney disease; DMT, dietary restriction plus medical therapy; RNA-seq, ribonucleic acid sequencing; ZDF, Zucker Diabetic Fatty; ZDSD, Zucker Diabetic Sprague Dawley.

Medications were administered at doses which have been shown to be renoprotective in monotherapy in rat models of hypertensive or diabetic renal injury [42–45]. Liraglutide was dose-titrated to a daily maximum dose of 1 mg/kg in ZDF rats and 0.2 mg/kg in ZDSD rats as previously described [46]. Liraglutide was titrated to a lower daily dose in ZDSD rats as their intact leptin signalling system renders them more sensitive to liraglutide-induced adipsia and food aversion [47, 48]. Body weights and mid-morning plasma glucose levels (Freestyle Optium Neo, Abbott Laboratories, Chicago, IL) were examined on a weekly basis before and after intervention. Animals were humanely killed (intraperitoneal overdose of sodium pentobarbital (200mg/kg) in the ZDF study; exsanguination under isoflurane anaesthaesia in the ZDSD study) after an 8-week post-intervention period. Cervical dislocation was performed to confirm humane killing. An overview of the study design, experimental timelines, and study endpoints is provided in Figure 1.

### Sham surgeries

The DMT intervention was designed as a bariatric mimetic intervention and studied alongside Roux-en-Y gastric bypass (RYGB) surgery in the ZDF and ZDSD studies. The impact of RYGB alone (ZDF) and RYGB in combination with medical treatment (ZDSD) has been reported on separately to the DMT intervention [14, 15]. To control for the impact of surgery on renal endpoints, sham surgeries were performed in SHAM and DMT-treated rats. A week prior to surgery, glycaemic control was optimized with daily subcutaneous injection of insulin degludec (Tresiba^®^, Novo Nordisk, Denmark) to achieve a fasting plasma glucose <12 mmol/L. Animals were anaesthetised with isoflurane (5% induction dose; 2% maintenance dose) and administered a pre-operative prophylactic antibiotic, enrofloxacin 5mg/kg s.c. (Baytril, Bayer). A midline laparotomy was performed followed by closure. Buprenorphine (Animalcare Limited) analgaesia was provided at 0.01-0.05 mg/kg s.c. every 6 hours for the first 2 post-operative days and as required thereafter.

### Biochemical analyses

Pre- and post-intervention urine samples were collected over a duration of 16 hours in both the ZDF and ZDSD experiments. In the ZDF study, urinary analytes were measured using an autoanalyser (Roche/Hitachi Cobas c501 modular analyser) and resulting values used to derive the albumin-to-creatinine ratio in μg/mg (uACR). In the ZDSD study, urinary concentrations of albumin were examined by enzyme-linked immunosorbent assay (ELISA) (K15162C Meso Scale Discovery, Rockville, MD) and expressed as the albumin excretion rate in μg/hour.

In the ZDF study, plasma total cholesterol (WAKO 294-65801) and triglycerides (Cayman Chemical 10010303) were measured at 8-week follow-up using colorimetric assays. In the ZDSD study, serum total cholesterol and triglycerides were measured at 8-week follow-up using an Atellica^®^ Solution Immunoassay and Clinical Chemistry Analyser (Siemens Healthineers).

### Histological and immunohistochemical analyses

Five to ten-micron thick sections of formalin-fixed paraffin-embedded kidney were used for haematoxylin and eosin (H&E) and immunohistochemical staining. ZDF kidney sections were stained with anti-Wilms’ tumour-1 (WT-1) antibody (C-19, sc-192 Santa Cruz Biotechnology; 1:250 dilution) using a Dako Autostainer Link 48 system. Signal amplification was achieved using the horseradish peroxidase-based FLEX system with signal development using 3,3’-diaminobenzidine (DAB) substrate. ZDSD kidney sections were stained with anti-ACOX1 antibody (ab184032, Abcam; 1:200 dilution) using the VECTASTAIN Elite ABC-HRP peroxidase kit (PK-6200, Vector Laboratories). IgG isotype controls (ADI-950-231-0025, Enzo Life Sciences) and no antibody controls were used to confirm the specificity of staining. All slides were digitized at 20x magnification using an Aperio AT2 Digital Slide Scanner (Leica Biosystems).

Scanned WT-1-stained sections were used to measure glomerular area in Aperio ImageScope (Leica Biosystems) in the ZDF experiment. Scanned H&E-stained sections were used to measure glomerular area in QuPath in the ZDSD experiment [49]. Glomerular volume was calculated from glomerular area using the Weibel and Gomez formula [50]. In both studies, thirty glomerular tufts per sample were manually annotated at random throughout the renal cortex, from a minimum of six animals per experimental group.

### Transmission electron microscopy

Glutaraldehyde-fixed and 1% osmium tetroxide post-fixed renal tissue was dehydrated and infiltrated with EPON^™^ Epoxy Resin. Ultra-thin sections were prepared and examined by transmission electron microscopy (TEM) (Tecnai^™^ G2 12 BioTWIN). Images were analyzed using ImageJ software (NIH, https://imagej.nih.gov/ij/). Data were acquired from glomeruli in both the ZDF and ZDSD studies (minimum of six animals per experimental group), while proximal tubular data were also acquired in the ZDSD experiment.

All glomerular ultrastructural measurements were recorded from six separate capillary loops, representative of at least three separate glomeruli per sample. Podocyte foot process frequency (PFPF) was measured at 9900x by determining the number of podocyte foot processes (FPs) per unit length (8 μm) of glomerular basement membrane (GBM). Six measurements were recorded per sample. GBM thickness was measured according to the Haas method at 20500x [51]. Podocyte foot process diameter (PFPD) was measured as a reciprocal of PFPF at 20500x. Twenty-four GBM thickness and PFPD measurements were recorded per specimen.

Mitochondrial roundness was assessed as a marker of mitochondrial stress and measured in thirty images of proximal tubular cells per animal, fifteen each from the pars convoluta and pars recta segments. The pars convoluta and pars recta sections of the proximal tubule were distinguished by their ultrastructural morphometry and mitochondria contained therein imaged separately [52, 53]. For each of the pars convoluta and pars recta sections, fifteen non-overlapping images from three distinct regions (five images/region) were acquired at 16500x per sample. Each image contained a minimum of five mitochondria for quantification. Images were captured in the basal region of pars convoluta cells for consistency, adjacent to the tubular basement membrane. Longer mitochondria which ran off the edge of the image, and for which less than 2 μm of their course was captured by the image window, were excluded from analysis. Mitochondrial parameters (area, perimeter, major and minor axis, roundness, and aspect ratio) were measured using the freehand line selection tool of ImageJ. Roundness is the inverse of the aspect ratio (major axis/minor axis length) and is calculated as:

$$\frac{4\times \;area}{\left(\pi \times {\;major\;axis}^{2}\right)}$$

### Transcriptomic and quantitative real-time PCR analyses

Ribonucleic acid (RNA) was extracted from renal cortical samples using an RNeasy Plus Mini Kit (Qiagen). RNA was also extracted from liver and epididymal fat pads in the ZDSD experiment. The concentration and purity of RNA samples were determined using a Nanodrop^™^ 2000 Spectrophotometer (Thermo Fisher Scientific) and RNA sample integrity assessed using Agilent RNA 6000 Nano kits (Agilent Technologies).

For renal cortical RNA-sequencing (RNA-seq) in the ZDF experiment, RNA library preparation was carried out using the TruSeq^®^ Stranded mRNA NeoPrep kit (Cat. No. NP-202-1001, Illumina). Libraries were sequenced on the Illumina NextSeq^®^ 500 platform in a paired-end fashion at a read length of 2x75bp. For renal cortical RNA-seq in the ZDSD experiment, RNA library preparation was carried out using the TruSeq^®^ Stranded Total RNA Library Prep Gold kit (Cat. No. 20020598, Illumina). Libraries were sequenced on the Illumina NovaSeq^®^ 6000 platform in a paired-end fashion at a read length of 2x100bp. Sequencing fastq files have been deposited in Gene Expression Omnibus (GEO) (accession numbers: GSE117380 for ZDF data; GSE169085 for ZDSD data).

For quantitative real-time polymerase chain reaction (qRT-PCR) analyses, samples were treated with deoxyribonuclease (DNase) 1 and complementary deoxyribonucleic acid (cDNA) synthesized using SuperScript^™^ II Reverse Transcriptase Kit (Invitrogen). Messenger RNA (mRNA) expression of *Acox1*, *Ehhadh*, *Acaa2*, and *Pdk4* was quantified in renal cortical, liver, and adipose tissue samples (TaqMan^®^ Gene Expression Assays, Thermo Fisher Scientific and QuantStudio 7 Flex System, Applied Biosystems). β-actin was used as the endogenous reference gene for all qRT-PCR analyses. Comparative analysis was performed using the ΔΔCt method [54], with healthy control animals (fa/+ or SD) serving as calibrators.

### RNA-seq bioinformatic analyses

The quality of the raw renal cortical RNA-seq fastq files was analyzed using FastQC (0.11.2) [55]. Quality filtering of reads was performed using Prinseq (0.20.3) for the ZDF data and using Trim Galore (0.4.0) for the ZDSD data [56, 57]. Adapter removal was not performed for the ZDF data due to low adapter content. Adapter removal was performed using Cutadapt (1.9) for the ZDSD data [58]. The data was mapped with STAR (2.5.2b) towards the rat reference genome, rn5 [59]. Read quantification was performed with HTSeq (0.6.1p1) for the ZDF data and with featureCounts (1.6.4) for the ZDSD data [60, 61].

Further analysis was performed using the R statistical programming language (4.1.1) [62]. Differential expression analysis was performed using DESeq2 [63]. The data was normalized by size factors. A negative binomial generalized linear model was fitted to the normalized data, with the Wald statistic used to identify differentially expressed genes (DEGs). The p-values were adjusted for multiple testing with the Benjamini-Hochberg procedure [64]. A regularized log (rlog) transformation was subsequently applied to gene expression counts.

Clustering by principal component analysis (PCA) was performed using rlog gene expression counts and plotted by experimental group using the R package factoextra [65]. Volcano plots were created using the R package ggplot2 [66, 67]. Differentially expressed transcripts between experimental groups, considered as those with an absolute fold-change ≥1.3 and adjusted p-value <0.05, were used as the input for functional enrichment analyses and enumerated on Venn diagrams using the R package ggvenn [68]. Pathways (Reactome database) and gene ontology (biological process and cellular component) terms over-represented between experimental groups in both animal models were examined using the function‘compareCluster’ from the R package clusterProfiler [69, 70]. Results of functional enrichment analyses were rendered using the R package clusterProfiler [69, 70]. Upstream regulator analysis was performed using Ingenuity PathwayAnalysis (IPA) [71], with results presented on a bubble plot generated using the R package ggplot2 [66, 67].

### *In silico* deconvolution of the predicted cellular source of DMT-responsive transcripts

A shared renal cortical transcriptomic response to DMT was defined by identifying genes which were differentially expressed (absolute fold-change ≥1.3, adjusted p-value <0.05) and sharing directionality of regulation (upregulated or downregulated) for the DMT vs SHAM comparison in both the ZDF and ZDSD models. To interrogate renal tubular epithelial cell localisation of these DMT-responsive transcripts commonly changed in both models, we accessed a publicly available proteomics dataset of 14 rat tubular epithelial cell types [72]. Transcripts commonly changed by DMT relative to SHAM in both models were intersected with the rat tubular epithelial cell protein expression matrix and plotted on a heatmap [73]. Line plots of cell-type expression of three PPARα-responsive proteins whose mRNA expression was induced by DMT, ACOX1, EHHADH, and ACAA2, were generated.

Further interrogation of the localisation of the DMT-responsive renal transcriptomic signature was performed by using a single-nucleus RNA sequencing (snRNA-seq) dataset containing samples from 3 individuals with early DKD [74]. Raw gene expression counts and cell assignments were analyzed according to standard single cell clustering workflows using the R package Seurat [75]. Assignment of cluster identity was cross-checked with the human diabetic kidney dataset on the Kidney Interactive Transcriptomics website, which is the same dataset analyzed herein. Average expression of each transcript across the defined cell types was calculated using the function ‘AverageExpression’. Transcripts commonly changed by DMT relative to SHAM in both models, after conversion to their human orthologs [76], were intersected with the human diabetic kidney gene expression matrix and plotted on a heatmap [73]. Violin plots of cell-specific expression of three PPARα-responsive transcripts whose mRNA expression was induced by DMT, *Acox1*, *Ehhadh*, and *Acaa2*, were generated with the Seurat function ‘VlnPlot’.

### *In silico* deconvolution of medication- and PPAR isotype-specific transcriptomic responses to DMT using a network pharmacology approach

Genes responsive to the medications fenofibrate, liraglutide, metformin, ramipril, and rosuvastatin (FLMRR) and to PPAR isotypes (alpha, beta/delta, and gamma) were obtained using IPA [71]. Separate lists of the five medications, alongside their corresponding cardinal drug targets, as well as the three PPAR isotypes were generated in IPA. For each list, a network was grown to identify target genes using the ‘Grow’ tool in the ‘Build’ section of the ‘My Pathways’ interface. Only experimentally observed relationships were permitted. Drug, chemical, disease, and function categories were excluded. The data underlying each network were exported from IPA and imported into RStudio [62]. The Entrez IDs of medication and PPAR target genes were converted to rat gene symbols [77, 78], and intersected with the list of DEGs commonly changed by DMT relative to SHAM in both animal models.

Doughnut plots were generated using the R package ggplot2 to illustrate the number and percentage of medication- and PPAR-responsive genes identified in IPA, stratified by medication type/PPAR isotype (inner layer) as well as presence in or absence from the DMT vs SHAM DEG list common to the ZDF and ZDSD models (outer layer) [66, 67]. Venn diagrams were created using the R package venn to illustrate the overlap and separation in transcripts responsive to each of the five medications and each of the three PPAR isotypes [79].

Medication- and PPAR-responsive transcripts contained within the common DMT vs SHAM DEG list were intersected with a rat tubular epithelial cell protein expression matrix [72]. Transcripts were classified as belonging to one of two categories, the proximal tubule or the rest of the renal tubule, based on site of maximal abundance following hierarchical clustering when plotted on heatmaps, with this information being extracted from the heatmap dendrograms [73]. Doughnut plots were subsequently generated using the R package ggplot2 to summarise the number and percentage of transcripts stratified by fenofibrate- or PPARα-responsiveness (inner layers), presence or absence from the tubular epithelial cell proteomics dataset (middle layers), and localisation in either the proximal tubule or the rest of the renal tubule (outer layers) [66, 67].

Functional enrichment analyses were performed on medication-responsive genes present in the common DMT vs SHAM DEG list, stratified by fenofibrate-responsiveness and renal tubular localisation (proximal tubule vs rest of tubule). Pathways (Reactome database) and gene ontology (cellular component) terms over-represented between these gene clusters were examined using the function ‘compareCluster’ from the R package clusterProfiler, with results presented as dotplots [69, 70]. A network plot of pathways and gene ontology terms associated with fenofibrate-responsive and proximal tubular abundant transcripts changed by DMT was generated using the function ’cnetplot’ from the R package enrichplot [80].

### Urinary NMR metabolomic analyses

Proton nuclear magnetic resonance (^1^H-NMR) spectroscopy was performed on timed urine samples obtained at baseline and at 4 weeks after intervention according to standard Bruker In Vitro Diagnostic Research (IVDr) methods [81]. Urine samples were thawed at room temperature for 20 min before a brief spin at 2000 g at 4 °C for 10 min. NMR samples for 5 mm SampleJet racks were prepared by mixing 9 parts urine with 1 part urine buffer (1.5 M KH_2_PO_4_ pD 6.95, 0.5% w/v NaN3, 0.1% w/v 3-trimethylsilyl propionic-2,2,3,3 acid sodium salt D4 (TSP-d4) in 99.8% D_2_O) using a SamplePro Tube L liquid handling robot (Bruker BioSpin, Ettlingen, Germany). The temperature was kept at 279 K throughout the sample preparation process. POM balls were added to tube caps in the finished sample tube rack before placing the rack in the cooled SampleJet sample changer on the spectrometer.

1D nuclear Overhauser effect spectroscopy (NOESY), 1D Carr-Purcell-Meiboom-Gill (CPMG) and 2D J-resolved experiments were acquired for each sample with a 600 MHz Bruker Avance III HD spectrometer at 300 K equipped with a 5 mm BBI room temperature probe, using the pulse sequences ‘noesygppr1d’, ‘cpmgpr1d’ and ‘jresgpprqf’, respectively, according to the IVDr SOP [81]. Urine samples were randomised during sample preparation and pooled samples containing aliquots of samples from each of the study groups were included as quality control steps. TSP-d4 was used for internal chemical shift referencing.

To facilitate metabolite annotation, a set of 2D experiments on four selected samples were acquired on an Oxford 800 MHz magnet equipped with a Bruker Avance III HD console, a 3 mm TCI CryoProbe and a cooled SampleJet sample changer. ^13^C-HSQCs were acquired using the pulse sequence ‘hsqcedetgpsisp2.3’ using spectral widths of 20 and 90 PPM in the direct and indirect dimensions, respectively, collecting 64 scans per increment for a total of 512 increments and 2048 data points. The acquisition time was 63.9 and 14 ms for the direct and indirect dimensions, respectively, and the relaxation delay was 1.5 s. ^1^H-^1^H-TOCSYs were acquired using the pulse sequence ‘dipsi2esgpph’ with sweepwidths in both dimensions of 12 PPM, collecting 16 scans per increment into 512 increments and 8192 data points. Acquisition times were 0.426 s and 26.6 ms for the direct and indirect dimensions, respectively. The TOCSY transfer delay was 60 ms and the relaxation delay between scans was 1 s. ^1^H-^1^H-COSYs were acquired with the pulse sequence ‘cosygpppqfpr’. Sweepwidths were 13.95 PPM in both dimensions; 4 scans per increment were collected to a total of 1024 increments and 2048 data points. The acquisition time was 92 ms and the relaxation delay 2 s. All 2D spectra were referenced to TSP-d4.

The 1D NOESY spectra destined for peak picking and multivariate analysis were zero-filled twice before Fourier transformation into 132k data points, including addition of 0.3 Hz exponential line-broadening and referencing to TSP-d4. Spectra were processed in TopSpin3.5pl7 (Bruker BioSpin, Ettlingen, Germany). The 1D NOESY spectra were loaded into Matlab using RBNMR [82]. Baseline correction of the spectra was performed due to the high urinary glucose concentrations present in untreated ZDSD rats using the command ‘msbackadj’ with window size set to 1000, quantile set to 0.1, and stepsize set to 500 [83].

### ^1^H-NMR spectral processing in the ZDSD model

Further analysis of NMR data was performed using the R statistical programming language (4.1.1) [62]. The 1D nuclear Overhauser effect spectroscopy (NOESY) spectra and parts per million (PPM) chemical shift values were imported and processed to a peak intensity matrix according to a standard workflow using the R package Speaq [84]. Peak detection was performed using a Mexican hat wavelet method implemented by the function ’getWaveletPeaks’. Detected peaks were aligned and grouped to a single PPM index value using the function ’PeakGrouper’. Illustrative plots of raw spectra, peak detection, and peak grouping/alignment for three peaks of interest, which were generated using the Speaq function ’ROIplot’, are presented in Supplementary Figure S1.

Silhouette values were calculated as a metric of the quality of peak grouping using the function ’SilhouetR’. Peak groupings with a silhouette value less than 0.6 were removed and the peaks regrouped with the function ’regroupR’ – this process was repeated iteratively until all peak groupings had a silhouette value ≥0.6. Peak filling was performed to detect peaks that may have been missed during the first round of peak detection. Finally, a peak intensity matrix was built with grouped peaks (identified by their PPM shift values) as columns and samples as rows. A probabilistic quotient normalisation (PQN) was applied to the peak intensity matrix [85, 86], which was subsequently used as the input for multivariate statistics. Annotation of processed spectra was performed using Chenomx 8.6 software (Chenomx Inc.), the Human Metabolome Database (HMDB), and the Biological Magnetic Resonance Data Bank (BMRB) [87–89].

### ^1^H-NMR clustering analyses and classification modelling in the ZDSD model

Clustering by PCA was performed using PQN-normalised NMR peak intensities and plotted by phenotype as biplots along principal components 1 and 2 using the R package factoextra [65].

The R package Multivariate methods with Unbiased Variable selection in R (MUVR) was used to fit a series of multivariate random forest (RF) classification models for comparison of two groups of samples at a time [90]. The models were fitted to PQN-normalised ^1^H-NMR peak intensity matrices (X matrices) containing the relevant samples for each comparison. Y response vectors indicating experimental group assignment were inputted to each supervised model. The MUVR algorithm minimises overfitting by performing recursive elimination of the least informative variables in a repeated double cross-validation procedure [90]. The following modelling parameters were used, as recommended: nOuter=5 (number of outer cross-validation segments, to ensure both classes were present in all model segments), nRep=100 (number of model repetitions), and varRatio=0.85 (proportion of variables maintained in the data per model iteration during variable elimination) [90].

The ’max’ RF model, which considers all relevant predictors without compromising classification performance [90], was selected to identify as many urinary ^1^H-NMR peaks relevant to classifying experimental group status as possible. The area under the receiver operating characteristic curve (AUC) and the number of misclassifications was used to assess model performance. Additional performance metrics relating to model classification, including sensitivity and specificity, were calculated by inputting actual sample class alongside predicted sample class at the 50% probability threshold (obtained from the MUVR model) to the function ‘confusionMatrix’ from the R package caret [91].

Mean decrease in Gini index was used to rank variable (urinary ^1^H-NMR peak) importance to RF model classification. Dotplots of selected ^1^H-NMR peaks ordered by mean decrease in Gini index were generated for each model using a custom ggplot2 plotting function [66, 67]. Mean decrease in Gini index values presented are the mean of estimates obtained from 100 model repetitions. PQN-normalised peak intensity by group was plotted for selected metabolites identified as important to RF models using ggplot2 [66, 67].

### Correlations between kidney structure, renal cortical transcripts, and urinary metabolites in the ZDSD model

Pearson correlation matrices between mean values for histological and ultrastructural parameters and rlog gene expression counts as well as PQN-normalised urinary ^1^H-NMR peaks from 4 weeks post-intervention were constructed on a per animal basis using the base R function ‘cor’ [62]. Transcriptomic pathway over-representation analysis was performed for the ZDSD DMT vs SHAM comparison using the R package ReactomePA [92]. Gene-structure correlations for genes belonging to enriched peroxisomal and mitochondrial FAO pathways between these two groups of rats were extracted. Metabolite-structure correlations for selected metabolites which were differentially abundant between ZDSD DMT and SHAM rats were extracted. Correlation matrices were plotted using the ggcorrplot R package [93].

A network plot was generated to illustrate the correlation structure between kidney structural parameters, renal cortical FAO transcripts, and urinary metabolites following DMT. A Pearson correlation data frame between all three components was constructed using the function ’correlate’ from the R package corrr [94]. Correlations with Pearson |r| <0.5 were removed. The correlation data frame was converted to an igraph network using the igraph function ’graph_from_data_frame’[95], and plotted with the ’stress’ layout using the R packages tidygraph and ggraph [96, 97]. Network nodes are clustered based on multidimensional scaling of Pearson correlation values.

### Descriptive and inferential statistics

ZDF and ZDSD study endpoints, statistical tests by which they were analysed, and location within the manuscript are presented in Supplementary Table S1. Percentage delta change in metabolic parameters (body weight and plasma glucose) and albuminuria (uACR for ZDF model, uAER for ZDSD model) was calculated as:

$$\left(\frac{\;Post-intervention\;value\;-\;Pre-intervention\;value\;}{\;Pre-\mathrm{int}ervention\;value\;}\right)\times 100$$

Statistical analyses were performed using the R package rstatix in RStudio (R version 4.1.1) [62, 98]. P-values were adjusted for the number of comparisons using the Benjamini-Hochberg method [64]. P<0.05 was considered statistically significant. Study endpoints are plotted as boxplots or violin plots using the R package ggplot2 [66, 67], with individual data points for each animal superimposed. For proximal tubular mitochondrial structural data, where per animal effect sizes are naturally smaller, individual annotations for all animals in each group are plotted to provide additional insight into data distribution [66, 67].

## Results

### Improvements in metabolic parameters following DMT in the ZDF and ZDSD studies

DMT resulted in mean reductions in body weight of 14.5±4.1% (ZDF) and 23.6±2.9% (ZDSD)(Table 1). DMT led to mean reductions in plasma glucose of 52.3±12.9% (ZDF) and 26.5±38.1% (ZDSD). Circulating triglycerides, but not cholesterol levels, were reduced by DMT across both models.

### Improvements in albuminuria and glomerular structure following DMT in the ZDF and ZDSD studies

**Figure 2:**
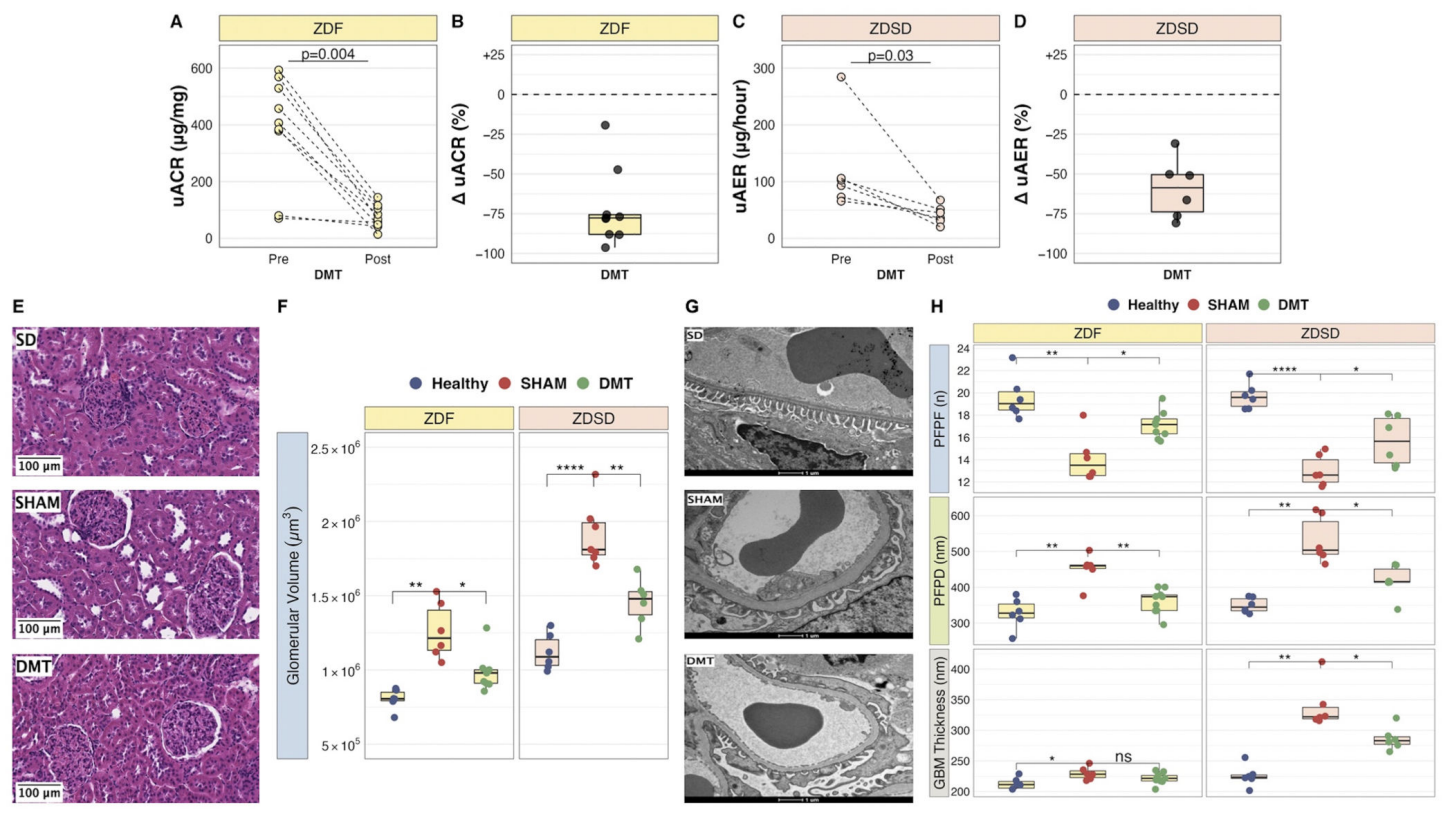
DMT attenuates glomerular injury in ZDF and ZDSD rats. A-B: Absolute uACR values before and after intervention (A) and percentage delta change in uACR values (B) in DMT-treated ZDF rats (n=9). C-D: Absolute uAER values before and after intervention (C) and percentage delta change in uAER values (D) in DMT-treated ZDSD rats (n=6). Statistical significance of differences in urinary albumin values before and after intervention was derived using Wilcoxon signed-ranked tests. E: Representative images (20x, scale bar 100 μm) of H&E-stained kidney sections from the three groups in the ZDSD experiment. F: Glomerular volume (μm^3^) values are plotted as boxplots with mean values per animal superimposed (ZDF: n=6 fa/+, n=6 SHAM, n=9 DMT; ZDSD: n=6 SD, n=7 SHAM, n=6 DMT). G: Representative images (9900x, scale bar 1μm) of TEM images of glomerular capillary loops from the three groups in the ZDSD experiment. H: PFPF (per 8 μm of GBM length), PFPD (nm), and GBM thickness (nm) were quantified using TEM images (ZDF: n=6 fa/+, n=6 SHAM, n=9 DMT; ZDSD: n=6 SD, n=6 SHAM, n=6 DMT). Six determinations of PFPF were made per animal, while 24 determinations of PFPD and GBM thickness were made per animal. Data are plotted as boxplots with mean values per animal superimposed. Statistical significance of between-group differences in glomerular structural parameters derived from multiplicity-corrected unpaired t-tests is denoted as follows: ns=not significant; *=p<0.05; **=p<0.01; ***=p<0.001; ****=p<0.0001. DMT, dietary restriction plus medical therapy; GBM, glomerular basement membrane; PFPD, podocyte foot process diameter; PFPF, podocyte foot process frequency; SD, Sprague Dawley; ZDF, Zucker Diabetic Fatty; ZDSD, Zucker Diabetic Sprague Dawley.

Baseline median [IQR] uACR was 7.4 [1.9] μg/mg in fa/+ heterozygous healthy control rats in the ZDF study. DMT reduced median [IQR] uACR values from 407.1 [150.7] μg/mg pre-intervention to 67.4 [56.9] μg/mg post-intervention in the ZDF model (p=0.004), corresponding to a -77.7 [12.4]% reduction in uACR (Figure 2A,B). Baseline median [IQR] uAER was 29.0 [13.5] μg/hour in Sprague Dawley healthy control rats in the ZDSD study. DMT reduced median [IQR] uAER values from 97.9 [27.3] μg/hour pre-intervention to 40.6 [17.3] μg/hour post-intervention in the ZDSD model (p=0.03), corresponding to a -58.6 [23.4]% reduction in uAER (Figure 2C,D).

Glomerular volume was elevated in SHAM rats relative to healthy control rats in both animal models (1.3×10^6^±1.9×10^5^vs 8.0×10^5^±7.0×10^4^μm^3^, p=0.001 in ZDF model; 1.9×10^6^± 2.1×10^5^ vs 1.1×10^6^±1.2×10^5^ μm^3^, p<0.001 in ZDSD model), and reduced following DMT (Figure 2E,F).

Mean podocyte foot process frequency (PFPF) was reduced in SHAM rats relative to healthy control rats in both animal models and increased following DMT (Figure 2G,H). Accordingly, mean podocyte foot process diameter (PFPD) was higher in SHAM rats relative to healthy control rats in both animal models and lower in DMT-treated animals (Figure 2G,H). Increases in GBM thickness were observed in SHAM rats relative to healthy control rats in both animal models, with the magnitude of increase being greater in the ZDSD model. GBM thickening was attenuated in DMT-treated ZDSD rats (Figure 2G,H).

### Improvements in proximal tubular mitochondrial morphology following DMT in the ZDSD model

**Figure 3:**
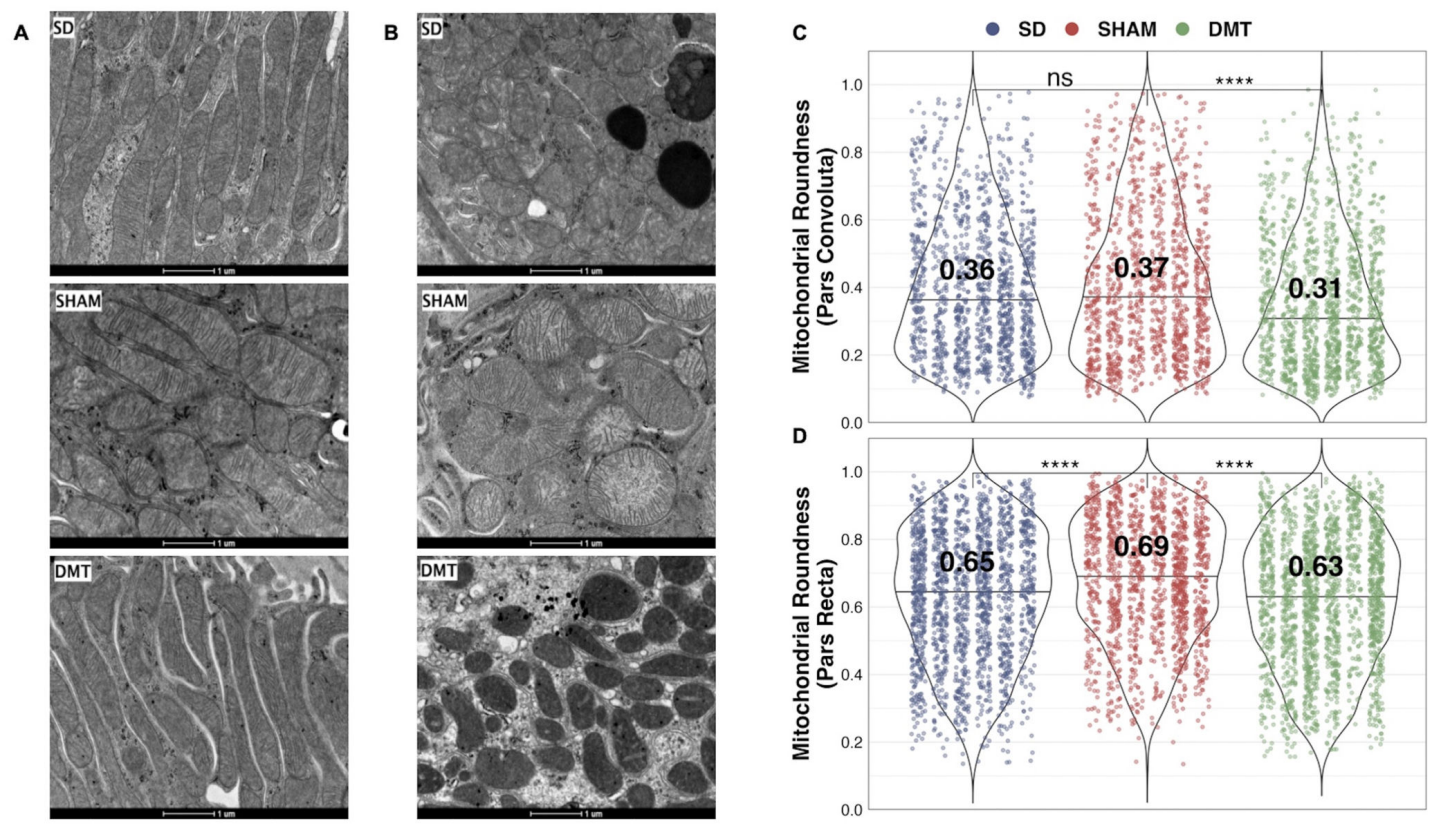
DMT improves proximal tubular mitochondrial morphology in ZDSD rats. A-B: Representative images (16500x, scale bar 1 μm) of TEM images of mitochondria in the pars convoluta (A) and pars recta (B) regions of the proximal tubule in the ZDSD study. C-D: Mitochondrial roundness in the pars convoluta (C) and pars recta (D) was quantified using TEM images (n=6 SD, n=6 SHAM, n=6 DMT). Mitochondria were quantified in 15 non-overlapping images captured from 3 distinct pars convoluta and pars recta regions (5 images/region) for each animal. Within each group, each animal is identified as its own column of dots. Median group values are printed on each violin. Statistical significance of between-group differences derived from multiplicity-corrected Wilcoxon rank-sum tests is denoted as follows: ns=not significant; *=p<0.05; **=p<0.01; ***=p<0.001; ****=p<0.0001. DMT, dietary restriction plus medical therapy; ZDSD, Zucker Diabetic Sprague Dawley.

As outlined in Supplementary Figure S2, in the ZDSD study, mitochondria in the pars convoluta had a greater two-dimensional area, were longer, and less round compared with mitochondria in the pars recta, as previously described [53]. In the pars convoluta, there was no difference in mitochondrial roundness between the SHAM and SD groups (Figure 3A,C). However, mitochondrial roundness did decrease in the pars convoluta of animals treated with DMT relative to the SHAM group. In the pars recta, mitochondrial roundness was increased in SHAM rats relative to the SD group (Figure 3B,D). Mitochondrial roundness in the pars recta was lower in DMT-treated rats relative to the SHAM group.

### Renal transcriptome profiling identifies enhanced FAO following DMT in ZDF and ZDSD rats

**Figure 4:**
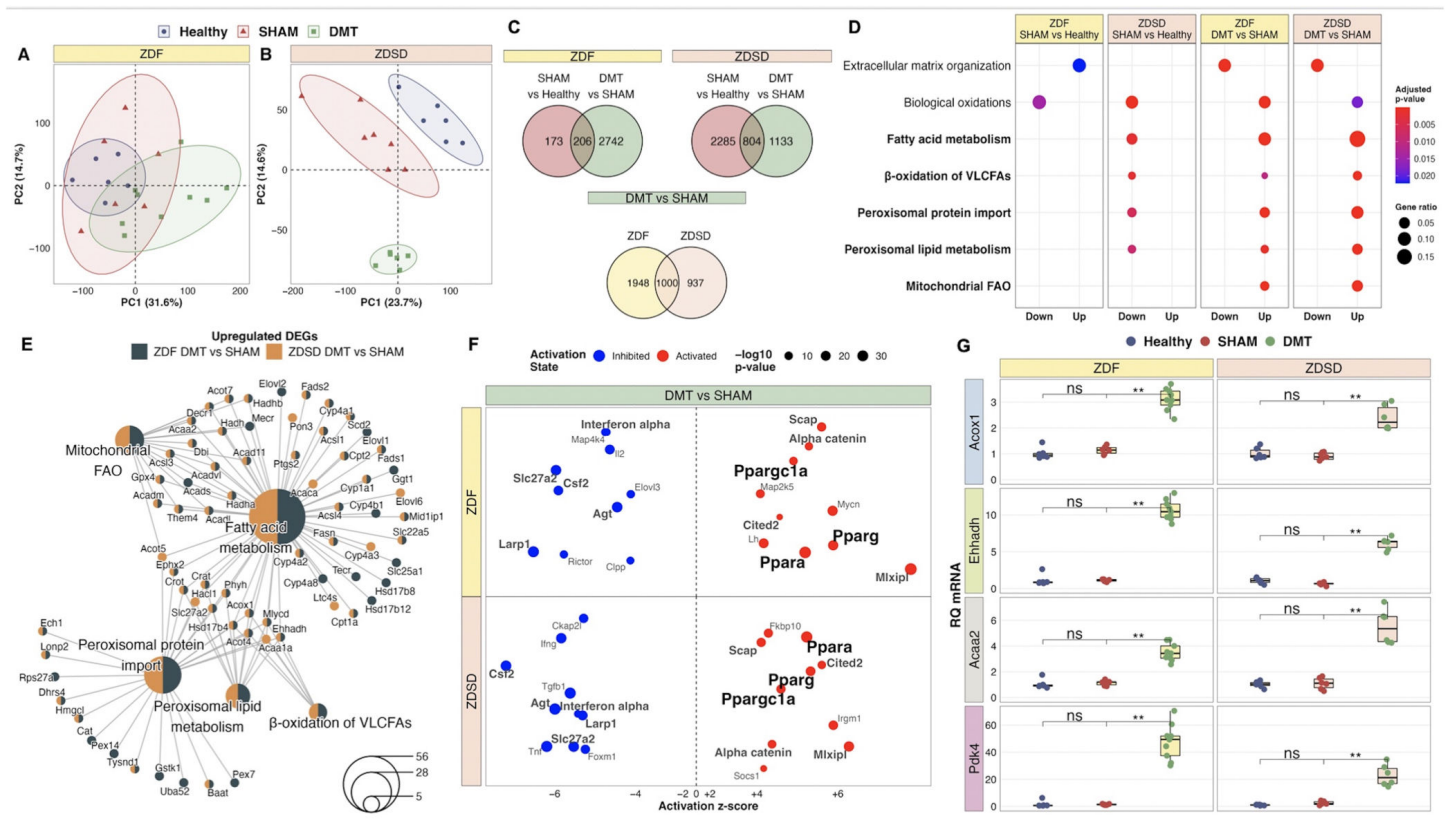
DMT induces a transcriptomic signature promoting FAO and opposing fibrosis in the renal cortex of ZDF and ZDSD rats. A-B: Principal component analysis of rlog gene expression counts in the ZDF (A) and ZDSD (B) experiments (ZDF: n=6 fa/+, n=6 SHAM, n=9 DMT; ZDSD: n=6 SD, n=7 SHAM, n=6 DMT). C: Venn diagram enumeration of the number of DEGs in the ZDF and ZDSD experiments, as well as the number of DEGs which were commonly changed in the DMT vs SHAM differential expression analysis in both studies. Transcripts with an absolute fold-change value ≥1.3 and adjusted p-value <0.05 were considered as differentially expressed. D: Dotplot of Reactome pathway over-representation analysis illustrating pathways commonly changed in the ZDF and ZDSD experiments, for both the SHAM vs Healthy and DMT vs SHAM comparisons [69, 70]. Pathways pertaining to fatty acid metabolism are bolded. E: Network plot of 5 fatty acid metabolism pathways changed by DMT in both animal models (bolded in panel D). Smaller gene nodes, changed by DMT relative to SHAM in either or both animal models, are connected to the larger Reactome pathway nodes to which they belong. Node size of pathways is scaled by the number of genes changed in that pathway (lower right legend). Pathway and gene nodes are coloured according to whether they are changed in the DMT vs SHAM differential expression analysis in one or both animal models. F: Bubble plot of predicted upstream regulators of DMT vs SHAM gene expression changes in both animal models, identified using IPA software [71]. Upstream regulator changes are presented for the DMT vs SHAM comparison in the ZDF and ZDSD models. The predicted status (inhibited or activated) of upstream regulators is plotted according to z-score. Dot size is scaled to reflect the statistical significance of z-score changes. Upstream regulators commonly changed in both models are bolded. G: qRT-PCR validation of expression changes in PPARα-responsive transcripts, both peroxisomal (*Acox1*, *Ehhadh*) and mitochondrial (Acaa2, Pdk4), in renal cortical tissue from the ZDF and ZDSD experiments (ZDF: n=6 fa/+, n=6 SHAM, n=9 DMT; ZDSD: n=6 SD, n=7 SHAM, n=6 DMT). Statistical significance of between-group differences derived from multiplicity-corrected Wilcoxon rank-sum tests is denoted as follows: ns=not significant; *=p<0.05; **=p<0.01; ***=p<0.001; ****=p<0.0001. DEG, differentially expressed gene; DMT, dietary restriction plus medical therapy; FAO, fatty acid oxidation; PC, principal component; RQ mRNA, relative quantification of messenger ribonucleic acid; VLCFA, very-long-chain fatty acid; ZDF, Zucker Diabetic Fatty; ZDSD, Zucker Diabetic Sprague Dawley.

Principal component analysis of kidney RNA-seq data identified discrete shifts across groups, and particularly following the DMT intervention, in both animal models (Figure 4A,B). DMT altered n=2,948 transcripts in the ZDF model and n=1,937 transcripts in the ZDSD model. Of transcripts altered between SHAM and healthy control rats (n=379 in ZDF model and n=3,089 in ZDSD model), n=206 (54.4%) were corrected by DMT in the ZDF model and n=804 (26.0%) were corrected by DMT in the ZDSD model (Figure 4C). In total, n=1,000 transcripts were commonly changed by DMT relative to SHAM in both models and shared directionality of regulation. Volcano plots emphasise that, in both models, there was induction of PPARα-responsive FAO transcripts (e.g., the peroxisomal enzyme *Ehhadh* and the mitochondrial enzyme *Pdk4*, which is a sensitive transcriptional marker of FAO [99]) following DMT (Supplementary Figure S3).

DMT downregulated fibrosis pathways (extracellular matrix organisation) while restoring biological oxidation capacity in both models (Figure 4D). Transcriptomic fibrosis pathways were upregulated in SHAM rats relative to healthy controls in the ZDF but not the ZDSD model, pointing to a more severe phenotype of renal injury in the ZDF model.

Enrichment of fatty acid metabolism pathways was a dominant transcriptomic response to DMT in both models (Figure 4D). In the ZDSD model, this contrasted with decreased fatty acid metabolism, and in particular peroxisomal lipid metabolism, observed in SHAM-operated rats. Peroxisomal and mitochondrial pathways upregulated by DMT are visualised in a network to illustrate the abundance of peroxisome proliferator-activated receptor-alpha (PPARα)-responsive transcripts (for example, *Acox1*, *Ehhadh*, *Acaa2*) causing FAO pathway enrichment (Figure 4E). Furthermore, the network visualisation of FAO pathways emphasises that many of the transcripts contributing to pathway over-representation were commonly changed in both models, and thus indicative of a common transcriptional response to DMT. Assessment of enriched biological process and cellular component gene ontology terms reinforced the downregulation of renal inflammation and fibrosis following DMT in both models, as well as the shared transcriptional response between both models resulting in induction of peroxisomal and mitochondrial fatty acid and long-chain fatty acid (LCFA) metabolism (Supplementary Figure S4A-D).

Activation of PPARα was predicted to increase FAO following DMT in both models by upstream regulator analysis (Figure 4F). We validated renal expression of peroxisomal (*Acox1*, *Ehhadh*) and mitochondrial (*Acaa2*, *Pdk4*) PPARα-responsive transcripts in both models by qRT-PCR (Figure 4G). PPARα-response genes were induced by DMT in the liver, the mammalian organ with the highest peroxisomal abundance [100], but not in visceral adipose tissue in the ZDSD model (Supplementary Figure S5). In particular, the upregulation of *Pdk4* following DMT in the renal cortex and liver points to increased FAO at these sites in response to the intervention [99].

### Peroxisomal and mitochondrial FAO transcripts induced by DMT predominantly map to the proximal tubule and are regulated by fenofibrate and PPARα

**Figure 5:**
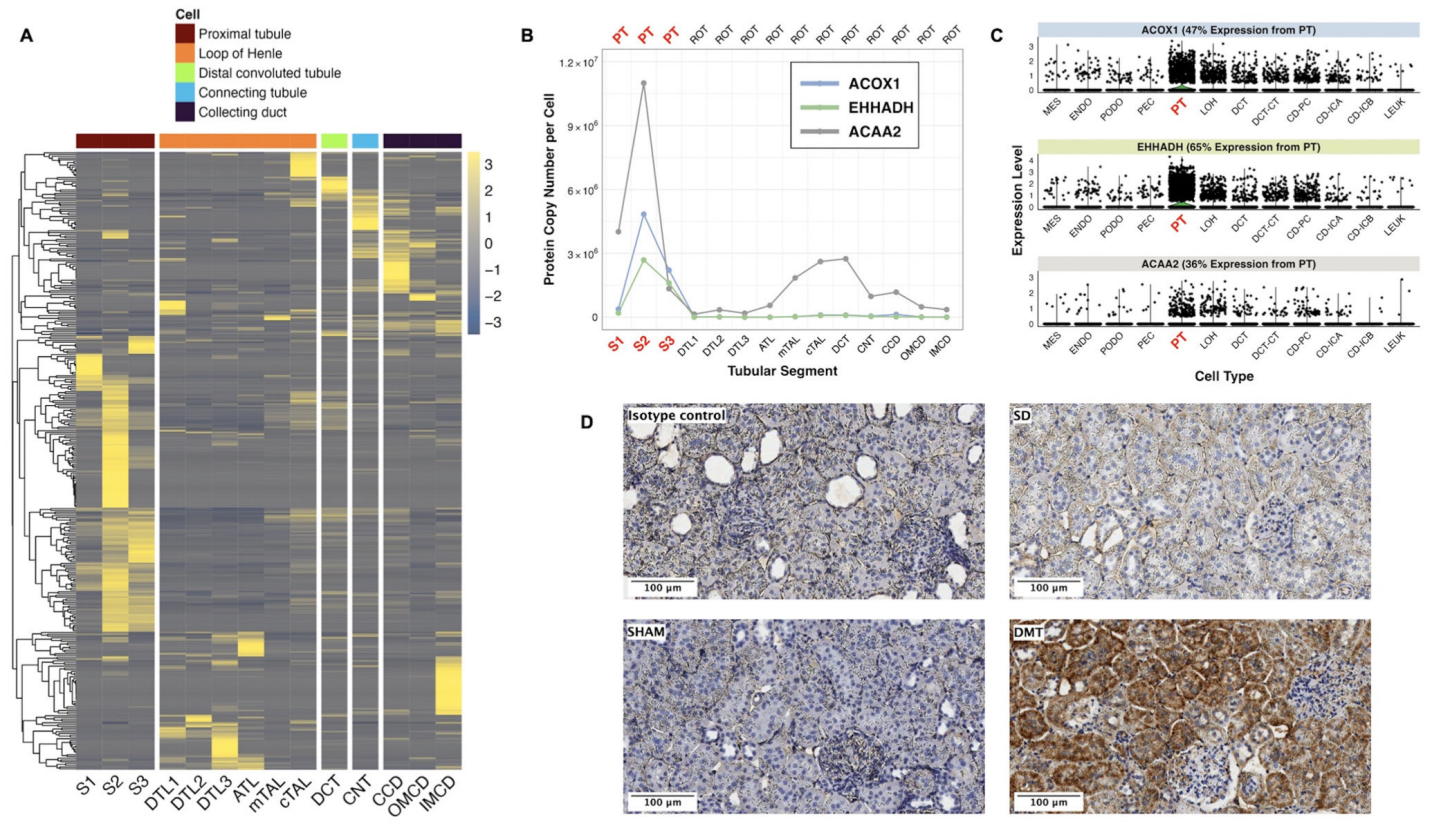
Identification of the proximal tubule as a key target of DMT in ZDF and ZDSD rats. A: DMT vs SHAM DEGs commonly changed and sharing directionality between both models were intersected with a proteomics dataset of microdissected SD rat kidney tubules [72]. Protein expression (copy number per cell) in rat tubular epithelial cells is plotted on the heatmap. Heatmap rows display DMT vs SHAM DEGs whilst each column represents 1 of 14 rat tubular epithelial cell types. B: Line plots of cell-specific expression in rat tubular epithelial cells of three PPARα-responsive transcripts upregulated by DMT. Tubular epithelial cell types are presented on the x-axis with protein expression (copy number per cell) in rat tubular epithelial cells on the y-axis. C: DMT vs SHAM DEGs commonly changed and sharing directionality between both models were intersected with a human diabetic kidney snRNA-seq dataset [74] to generate violin plots of cell-specific expression patterns of three PPARα-responsive transcripts upregulated by DMT. The 12 identified kidney cell types are presented on the x-axis with relative transcript expression levels in the human diabetic kidney on the y-axis. D: Representative images (20x, scale bar 100 μm) of kidney immunohistochemical validation of the proximal tubular localisation and upregulation of ACOX1 in DMT-treated rats in the ZDSD model. ATL, ascending thin limb of Henle’s loop; CCD, cortical collecting duct; CD-ICA, collecting duct-intercalated cell type A; CD-ICB, collecting duct-intercalated cell type B; CD-PC, collecting duct-principal cell; CNT, connecting tubule; cTAL, cortical thick ascending limb; DCT, distal convoluted tubule; DCT-CT, distal convoluted tubule-connecting tubule; DMT, dietary restriction plus medical therapy; DTL1, descending thin limb of Henle’s loop, short-loop; DTL2, descending thin limb of Henle’s loop, long-loop, outer medulla; DTL3, descending thin limb of Henle’s loop, long-loop, inner medulla; ENDO, endothelial cell; IMCD, inner medullary collecting duct; LEUK, leukocytes; LOH, loop of Henle; MES, mesangial cell; mTAL, medullary thick ascending limb; OMCD, outer medullary collecting duct; PEC, parietal epithelial cell; PODO, podocyte; PPARα, peroxisome proliferator-activated receptor-alpha; PT, proximal tubule; S1-S3, S1-S3 regions of the proximal tubule; SD, Sprague Dawley; ZDF, Zucker Diabetic Fatty; ZDSD, Zucker Diabetic Sprague Dawley.

We assessed cell type-specific expression patterns of DMT vs SHAM DEGs commonly changed in both models in a human diabetic kidney snRNA-seq dataset and in a rat tubular epithelial cell proteomics dataset [72, 74]. Many of these transcripts mapped to the proximal tubule of the human diabetic kidney (Supplementary Figure S6), and in particular to the S2 and S3 segments of the rat kidney (Figure 5A). Included amongst these proximal tubular abundant transcripts changed by DMT were PPARα-responsive genes with roles in peroxisomal (*Acox1*, *Ehhadh*) and mitochondrial (*Acaa2*) FAO (Figure 5B,C). The proximal tubular enrichment of ACOX1 as well as its induction following DMT was validated by immunohistochemistry in the ZDSD model (Figure 5D).

**Figure 6:**
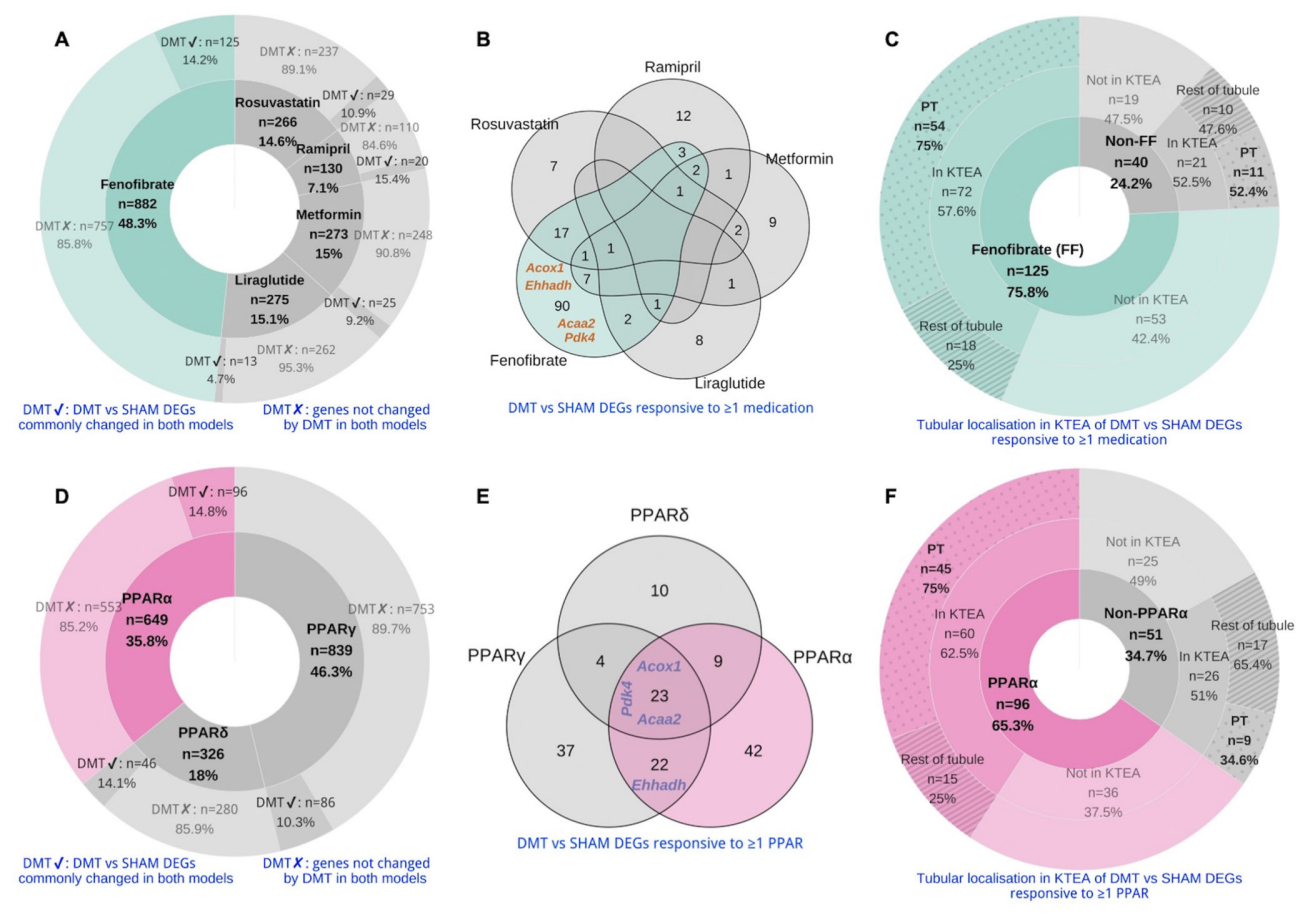
Identification of fenofibrate and PPARα as the dominant transcriptomic effectors of FAO following DMT in ZDF and ZDSD rats. A, D: Genes responsive to FLMRR medications (A) and PPAR isotypes (D) were retrieved from IPA, intersected with DMT vs SHAM DEGs commonly changed and sharing directionality between the ZDF and ZDSD models, and presented on doughnut plots with counts and percentages of transcripts in each category outlined. The inner doughnut layers outline all transcripts known to be responsive to each medication (A) or PPAR isotype (D). The outer layers stratify transcripts by presence in or absence from the DMT vs SHAM DEG list common to both models. B, E: Venn diagram enumeration of commonly changed DMT vs SHAM DEGs which are responsive to one or more of the FLMRR medications (B) or one or more of the PPAR isotypes (E). The localisation of selected PPAR-responsive transcripts is outlined on the Venn diagrams. C, F: Commonly changed DMT vs SHAM DEGs found to be responsive to FLMRR medications (C) or PPAR isotypes (F) are presented on doughnut plots with counts and percentages of transcripts in each category outlined. Transcripts are stratified by fenofibrate- (C) or PPARα- (F) responsiveness (inner layers), presence or absence from KTEA (middle layers), and localisation in either the proximal tubule or the rest of the renal tubule (outer layers). DEG, differentially expressed gene; DMT, dietary restriction plus medical therapy; FF, fenofibrate; FLMRR, fenofibrate, liraglutide, metformin, ramipril, and rosuvastatin; KTEA, Kidney Tubules Expression Atlas;PPAR, peroxisome proliferator-activated receptor; PT, proximal tubule; ZDF, Zucker Diabetic Sprague Dawley; ZDSD, Zucker Diabetic Sprague Dawley.

Using a network pharmacology approach, we assessed the magnitude and tubular epithelial cell localisation of medication- and PPAR isotype-specific transcriptomic responses in rats treated with DMT. Fenofibrate-responsive genes constituted the majority of medication-responsive genes present in the common DMT vs SHAM DEG list, equating to n=125 genes or 14.2% of all genes known to be changed by fenofibrate (Figure 6A). Overall, 165 of the common DMT vs SHAM DEGs were found to be responsive to one or more of the five medications in the FLMRR combination, with 125 (75.8%) of these being fenofibrate-responsive, including *Acox1*, *Ehhadh*, *Acaa2*, and *Pdk4* (Figure 6B). Of the medication-responsive transcripts present in both the common DMT vs SHAM DEG list and a rat tubular epithelial cell proteomics dataset [72], 75% of the fenofibrate-responsive transcripts were found to be proximal tubular-abundant, compared with 52% of transcripts responsive to FLMRR medications other than fenofibrate (Figure 6C).

Similarly, compared with other PPAR isotypes, there was a greater number of PPARα-responsive genes present in the common DMT vs SHAM DEG list, both in absolute numbers (n=96 transcripts) and as a proportion of all genes known to be changed by PPARα (14.8%; Figure 6D). Overall, 147 genes in the common DMT vs SHAM DEG list were found to be responsive to one or more PPAR isotypes, with 96 (65.3%) of these being PPARα-responsive, including *Acox1*, *Ehhadh*, *Acaa2*, and *Pdk4* (Figure 6E). Of the PPAR isotype-responsive transcripts present in both the common DMT vs SHAM DEG list and a rat tubular epithelial cell proteomics dataset [72], 75% of the PPARα-responsive transcripts were found to be proximal tubular-abundant, compared with only 34.6% of transcripts responsive to either PPARδ or PPARγ, but not PPARα(Figure 6F).

**Figure 7:**
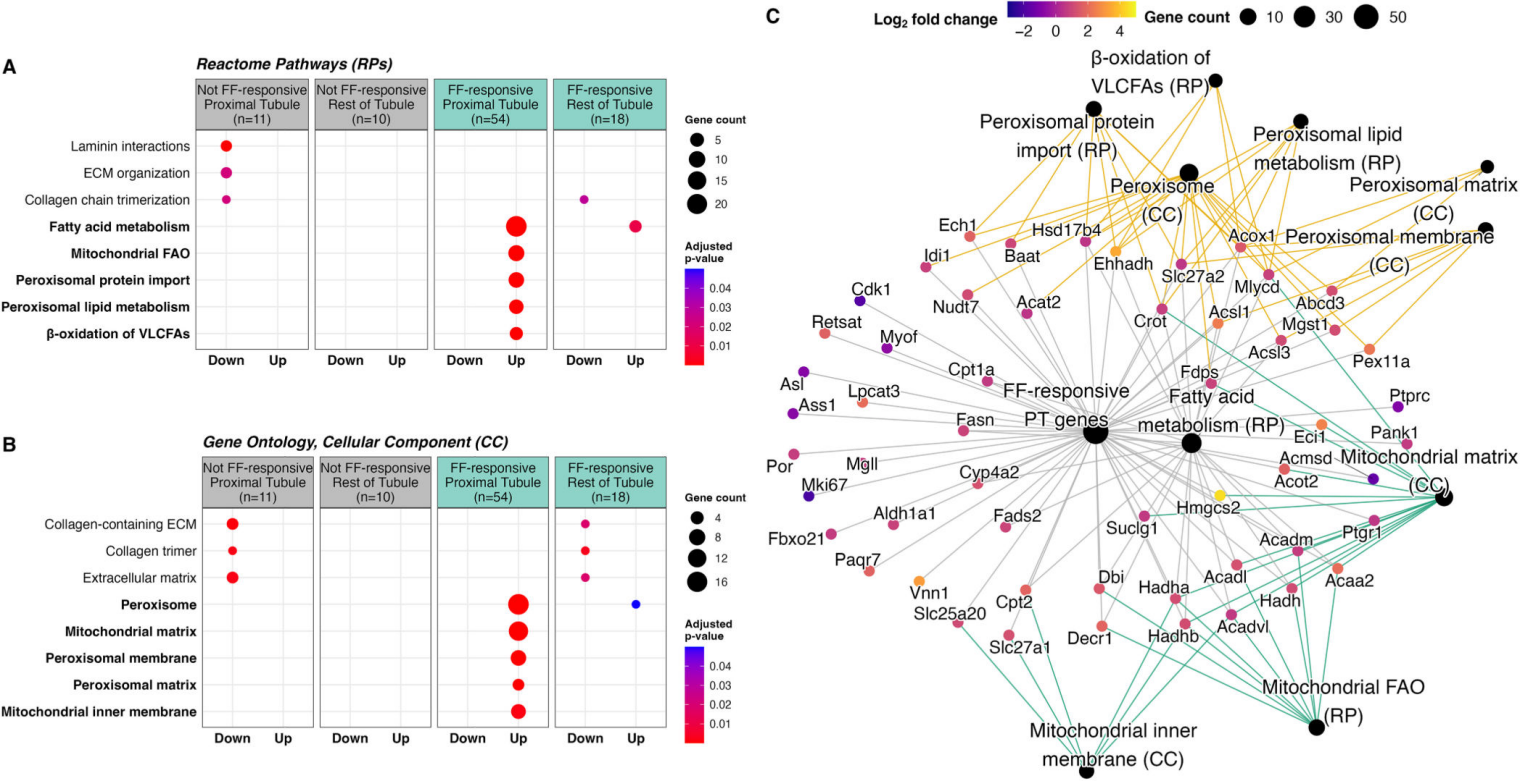
Functional enrichment of DMT vs SHAM DEGs according to fenofibrate-responsiveness and tubular epithelial cell localisation. A-B: DMT vs SHAM DEGs found to be responsive to one or more FLMRR medications were classified according to fenofibrate-responsiveness and predominant localisation in either the proximal tubule or the rest of the renal tubule (Figure 6C). Functional enrichment analyses (Reactome pathway analysis (A) and gene ontology cellular component testing (B)) were performed on medication-responsive DMT vs SHAM DEGs according to this classification system, with results presented on dotplots [69, 70]. Pathways pertaining to fatty acid metabolism and cellular components relating to peroxisomal and mitochondrial metabolism are bolded. C: Network plot of DMT vs SHAM DEGs which are fenofibrate-responsive and abundant in proximal tubular cells. Fatty acid metabolism pathways (bolded in panel A) and cellular components (bolded in panel B) enriched by these genes are overlaid on the network. Node size of pathways and cellular component terms is scaled by the number of genes belonging to those categories. Gene nodes are coloured according to log_2_ fold-change values for the DMT vs SHAM comparison, averaged between the ZDF and ZDSD models. Mitochondrial and peroxisomal nodes are identified by green and gold edges, respectively. CC, cellular component; DEG, differentially expressed gene; DMT, dietary restriction plus medical therapy; ECM, extracellular matrix; FAO, fatty acid oxidation; FF, fenofibrate; FLMRR, fenofibrate, liraglutide, metformin, ramipril, and rosuvastatin; KTEA, Kidney Tubules Expression Atlas; PPAR, peroxisome proliferator-activated receptor; PT, proximal tubule; RP, Reactome pathway; VLCFA, very-long-chain fatty acid; ZDF, Zucker Diabetic Sprague Dawley; ZDSD, Zucker Diabetic Sprague Dawley.

Amongst medication-responsive DMT vs SHAM DEGs present in a publicly available rat tubular epithelial cell proteomics dataset (n=93) [72], gene clusters were defined on the basis of proximal tubular abundance and fenofibrate-responsiveness. Functional enrichment analyses of these gene clusters using the Reactome database (Figure 7A) and gene ontology cellular component terms (Figure 7B) confirmed that proximal tubular abundant and fenofibrate-responsive transcripts were primarily responsible for induction of peroxisomal and mitochondrial FAO observed following DMT. Thus, fenofibrate was found to be the dominant medication effector of gene expression changes following DMT, and via its molecular target PPARα, contributed to induction of peroxisomal and mitochondrial FAO in the proximal tubule. A network visualisation of proximal tubular abundant and fenofibrate-responsive genes changed by DMT (n=54) illustrates individual transcripts mapping to peroxisomal and mitochondrial pathways and cellular component terms, as well as their strong induction following DMT (Figure 7C).

### DMT increases urinary PPARα-responsive nicotinamide metabolites and reverses DKD-associated changes in TCA cycle and clearance metabolite excretion

**Figure 8:**
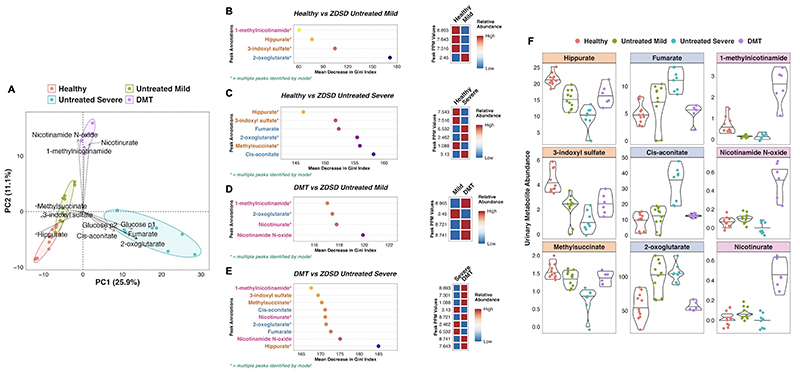
DMT is associated with urinary metabolite profiles consistent with PPARα activation and improvements in renal clearance and cellular energetics in ZDSD rats. A: PCA biplot of PQN-normalised urinary ^1^H-NMR peaks obtained before and at 4 weeks after intervention. Baseline and follow-up samples from SD rats were considered together given the lack of change in metabolomic profiles evident in this group. Samples from SHAM rats at baseline and follow-up were considered alongside baseline samples from DMT rats, and collectively designated as untreated ZDSD rats. Untreated ZDSD rats clustered based on disease severity into two subphenotypes, mild and severe, rather than by timing of sampling or by experimental group assignment. DMT refers to post-intervention values in this group. Arrows indicate loading vectors of metabolites driving separation of the groups along PCs 1 and 2. Healthy, n=11; untreated mild, n=11; untreated severe, n=8; DMT, n=6. B-E: Dotplots of urinary ^1^H-NMR peaks ranked by importance to performance of RF classification models for the following comparisons [90]: Healthy vs Untreated Mild (B), Healthy vs Untreated Severe (C), DMT vs Untreated Mild (D), and DMT vs Untreated Severe (E). Peaks are displayed in descending rank order from left to right according to the variable importance metric, mean decrease in Gini index. Variable importance estimates are mean values derived from 100 model repetitions. Metabolites which are efficiently cleared by the kidney (‘clearance metabolites’), TCA cycle intermediates, and PPARα biomarker metabolites involved in nicotinamide metabolism are highlighted in orange, blue, and pink bolded font, respectively. The adjacent heatmap illustrates relative abundance of metabolites based on mean peak intensity across all samples in the two groups evaluated by the RF model. F: PQN-normalised abundance by group of kidney clearance metabolites, TCA cycle intermediates, and PPARα biomarker metabolites identified by RF models in panels B-E (healthy, n=11; untreated mild, n=11; untreated severe, n=8; DMT, n=6). The first column reflects metabolites which are normally efficiently cleared by the kidney (orange panels), while the second and third columns reflect TCA cycle intermediates (blue panels) and PPARα biomarker metabolites (pink panels), respectively. Illustrative examples of the raw spectra and spectral processing for 1 peak from each group are presented in Supplementary Figure S1. NMR characteristics of the peaks are presented in Supplementary Table S2. ^1^H-NMR, proton nuclear magnetic resonance spectroscopy; DMT, dietary restriction plus medical therapy; p1, peak 1; p2, peak 2; PC, principal component; PCA, principal component analysis; PQN, probabilistic quotient normalisation; ZDSD, Zucker Diabetic Sprague Dawley.

Urinary metabolomic profiles of SHAM rats at baseline and follow-up clustered alongside baseline samples from DMT rats, and were collectively designated as untreated ZDSD rats (Figure 8A). Untreated ZDSD rats separated into two subphenotypes, mild and severe, based on urinary metabolomic changes relating to disease severity. The major sources of variation along principal components 1 and 2 pertained to a healthy SD control phenotype, an untreated severe ZDSD rat phenotype, and a post-DMT phenotype.

Loading vectors in the principal component analysis biplot indicate that increased levels of protein-bound solutes which are normally cleared by the kidney with high efficiency by tubular secretion (‘clearance metabolites’: 3-indoxyl sulfate, hippurate, and methylsuccinate) characterised the healthy SD phenotype (Figure 8A) [101]. Increased urinary excretion of glucose and TCA cycle intermediates (cis-aconitate, fumarate, and 2-oxoglutarate) characterised the untreated severe ZDSD rat phenotype. Given that these rats clustered away from other untreated ZDSD rats on the basis of increased urinary excretion of glucose and TCA cycle intermediates, they were designated as having a more severe phenotype, while those with lower urinary abundance of these metabolites were designated as having a mild phenotype. The post-DMT urinary metabolome was characterised by increased abundance of PPARα-responsive metabolites involved in nicotinamide metabolism (1-methylnicotinamide, nicotinamide N-oxide, and nicotinurate). Notably, changes in the urinary excretion of metabolites reflective of host-gut bacterial co-metabolism, which are well described following RYGB and associated with beneficial metabolic and renal effects of the procedure [102–104], were not pronounced following DMT.

A series of RF models effectively classified rats according to experimental group based on urinary metabolomic profiles. Metabolites reflective of renal clearance, TCA cycle intermediates, and PPARα-responsive nicotinamide metabolites were generally important to model performance (Figure 8B-E). Compared with SD rats, the ZDSD untreated mild and severe phenotypes were characterised by reduced urinary excretion of metabolites reflective of renal clearance and increased urinary excretion of TCA cycle intermediates, with these changes being more pronounced in the untreated severe phenotype (Figure 8B,C,F). Increased urinary excretion of PPARα-responsive nicotinamide metabolites occurred following DMT, alongside reversal of DKD-associated reductions in urinary clearance metabolites and increases in urinary TCA cycle intermediates which occurred in the ZDSD untreated mild and severe groups (Figure 8D-F). NMR characteristics of clearance metabolites, TCA cycle intermediates, and nicotinamide metabolites are provided in Supplementary Table S2.

### Renal FAO transcripts and urinary metabolites correlate with improvements in indices of glomerular and proximal tubular injury

**Figure 9:**
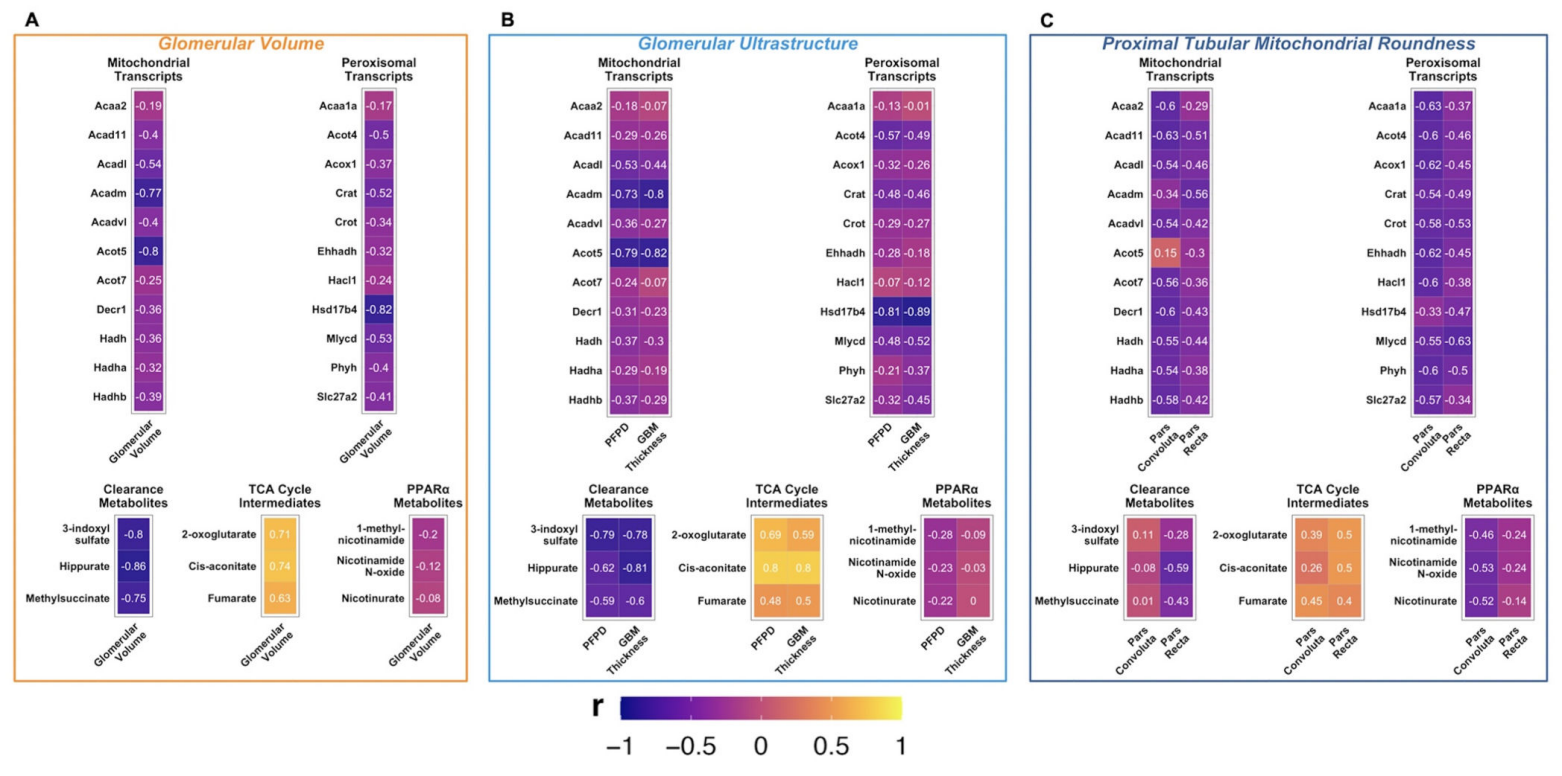
Molecular morphometric correlates of the renal response to DMT in ZDSD rats. A-C: Correlation plots highlighting Pearson correlation r values for changes in glomerular volume (A), glomerular ultrastructural parameters (B), and proximal tubular mitochondrial roundness (C) with gene expression changes and urinary metabolite abundance. Regularized log-transformed gene expression counts were used for gene-structure correlations. Selected transcripts which resulted in enrichment of peroxisomal and mitochondrial lipid metabolism pathways in ZDSD DMT rats relative to SHAM rats are plotted (also highlighted in the network plot in Figure 4E). PQN-normalised urinary ^1^H-NMR peaks from samples obtained at 4 weeks after intervention were used for metabolite-structure correlations. Correlations for metabolites which are efficiently cleared by the kidney (‘clearance metabolites’), TCA cycle intermediates, and PPARα biomarker metabolites involved in nicotinamide metabolism, many of which were differentially abundant between DMT-treated and untreated ZDSD rats (outlined in Figure 8F), are plotted. Individual cells in the correlation plots are scaled by colour indicating strength and directionality of the correlation. DMT, dietary restriction plus medical therapy;GBM, glomerular basement membrane; PFPD, podocyte foot process diameter; PPARα, peroxisome proliferator-activated receptor-alpha; PQN, probabilistic quotient normalisation; TCA, tricarboxylic acid; ZDSD, Zucker Diabetic Sprague Dawley.

Induction of peroxisomal and mitochondrial FAO transcripts in the renal cortex was inversely correlated with indices of glomerular and proximal tubular injury in the ZDSD model, with the strength of correlations being generally stronger for indices of proximal tubular than for glomerular injury (Figure 9A-C). Urinary PPARα biomarker metabolites were not significantly correlated with histological or ultrastructural indices of glomerular injury (Figure 9A,B), but were moderately correlated with proximal tubular mitochondrial roundness, particularly in the pars convoluta (Figure 9C). Renal cortical FAO transcripts, urinary PPARα biomarker metabolites, and mitochondrial roundness in the pars convoluta of the proximal tubule were strongly correlated with each other, clustering together on the relational network map of multi-dimensional correlations in Supplementary Figure S7.

Urinary excretion of metabolites reflective of renal clearance and TCA cycle intermediates was strongly correlated with glomerular but not proximal tubular injury (Figure 9A-C). TCA cycle intermediates clustered more closely to glomerular than proximal tubular injury parameters in the relational network map of multi-dimensional correlations (Supplementary Figure S7).

## Discussion

**Figure 10:**
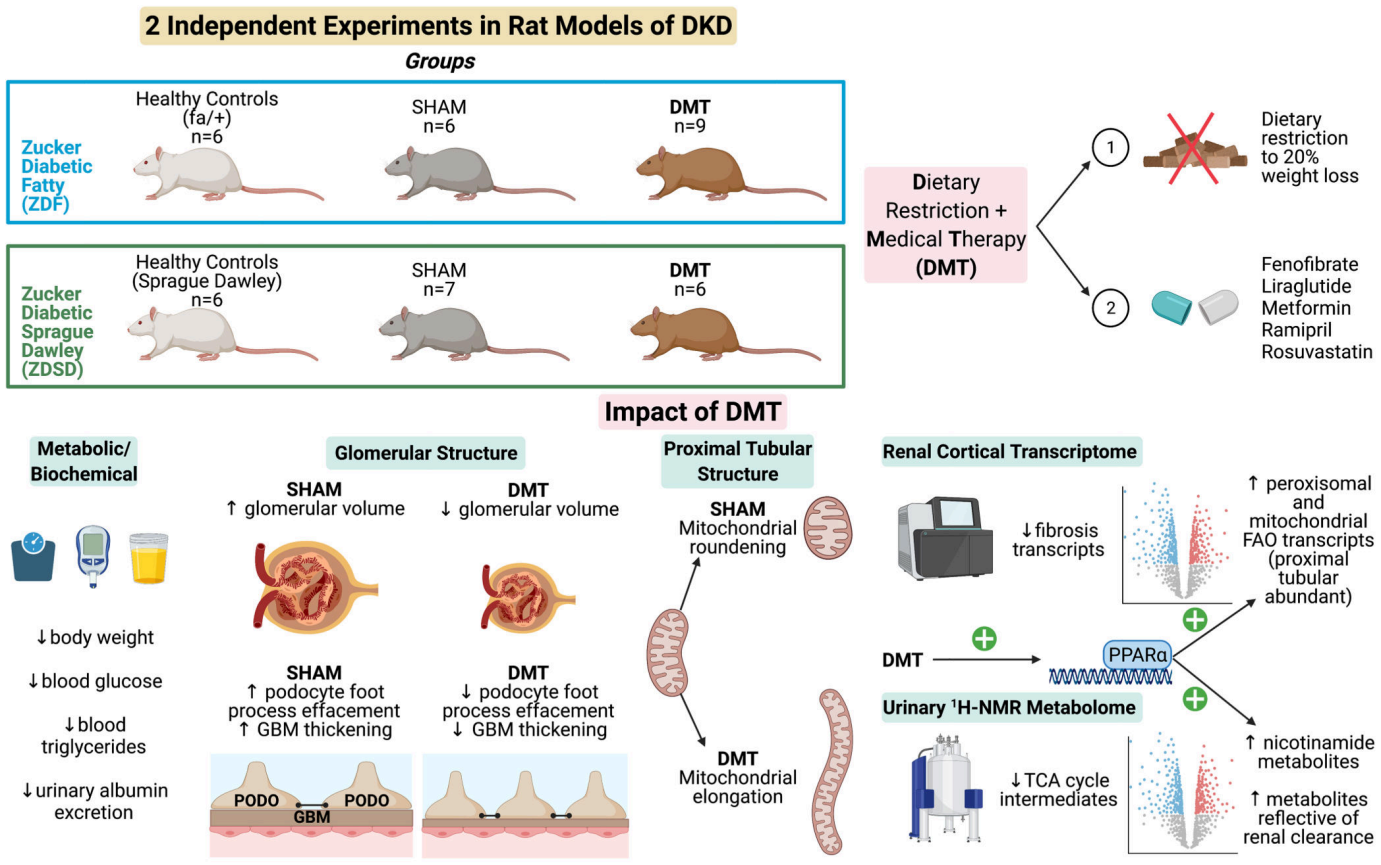
Overview schematic for the ZDF and ZDSD DMT preclinical studies. Created with BioRender.com. ^1^H-NMR, proton nuclear magnetic resonance spectroscopy; DKD, diabetic kidney disease; DMT, dietary restriction plus medical therapy; FAO, fatty acid oxidation; GBM, glomerular basement membrane; PODO, podocyte; PPARα, peroxisome proliferator-activated receptor-alpha; TCA, tricarboxylic acid; ZDF, Zucker Diabetic Fatty; ZDSD, Zucker Diabetic Sprague Dawley.

Dietary restriction plus medical therapy (DMT) elicited a strong renoprotective effect in both the ZDF and ZDSD rat models of DKD (Figure 10). Transcriptomic and metabolomic analyses result in two firm conclusions; 1) PPARα-driven increases in the expression of genes involved in FAO dominate the transcriptomic response and 2) said changes can be localised to the proximal tubule. Renal FAO transcripts and related urinary PPARα-responsive nicotinamide and TCA cycle metabolites were moderately-to-strongly correlated with observed improvements in glomerular and proximal tubular injury, implicating increased renal cortical PPARα activity and bioenergetic changes following DMT with the kidney structural improvements observed. A distinct strength of the presented findings is that renal responses to DMT were characterised in two independent experiments conducted in two different rat models of DKD. The shared renal cortical transcriptional response to the DMT intervention across models increases confidence in the reproducibility of the findings as well as the biological importance of proximal tubular peroxisomal and mitochondrial FAO as a mediator of reduced renal injury following DMT. Kidney and liver are the the two organs with the highest abundance of peroxisomes [100], and the the marked induction of genes relating to peroxisomal FAO in renal and hepatic tissue following DMT suggests that peroxisomal activation may be an important element of the therapeutic response [105].

In the ZDSD model, urinary abundance of TCA cycle intermediates increased in SHAM rats and decreased following DMT. Increased urinary excretion of TCA cycle intermediates has previously been reported in preclinical models of DKD [106, 107]. Renal cortical FAO transcripts were inversely correlated with the TCA cycle intermediates 2-oxoglutarate, fumarate, and cis-aconitate, suggesting that their reduction may reflect increased consumption in the TCA cycle as a consequence of enhanced renal cortical FAO [106]. Furthermore, the strong correlations between TCA cycle intermediates and indices of glomerular injury provides a functional link between FAO induction, TCA cycle activity, and improvements in glomerular structure following DMT.

DKD-associated reductions in the urinary excretion of 3-indoxyl sulfate, hippurate, and methylsuccinate were reversed by DMT. In a study employing untargeted mass spectrometry of urine and plasma in healthy controls and patients on dialysis, these 3 metabolites were part of a panel of 13 endogenous solutes which were identified as being highly efficiently cleared by the kidney through a combination of high plasma protein binding and tubular secretion [101]. Reduced clearance of hippurate was independently associated with increased risk of death in patients with CKD [108]. Thus, DKD-associated reductions in urinary excretion of these metabolites may reflect impaired proximal tubular functioning, specifically secretion by organic acid transporters, which was restored by the DMT intervention [101, 108].

Importantly, 3-indoxyl sulfate, hippurate, and methylsuccinate were strongly correlated with indices of glomerular injury in the ZDSD study. Indoxyl sulfate induces mesangial cell proliferation and podocyte injury, thereby contributing to glomerular injury [109–113]. Hippurate is a normal part of the endogenous urinary metabolite profile [114] and reduced urinary excretion of hippurate has been noted in the setting of kidney disease in humans [114–116] and in the obese Zucker rat model [117]. Hippurate has been proposed as a biomarker of mitochondrial function [114]. The strong correlations observed between both hippurate and TCA cycle intermediates with glomerular structural parameters may reflect impaired mitochondrial functioning that is corrected by DMT.

Reduced urinary excretion of methylsuccinate has also previously been reported in preclinical models of renal injury, specifically following administration of aristolochic acid and D-serine [118, 119]. Reduced urinary excretion of methylsuccinate following administration of D-serine provides a link to proximal tubular peroxisomal function as D-serine is metabolised by peroxisomes in proximal tubular cells and causes selective necrosis of the proximal straight tubule [119], the site of greatest peroxisomal abundance in the rat proximal tubule [52, 120]. Furthermore, methylsuccinate is a dicarboxylic acid, and peroxisomes are important for metabolism of dicarboxylic acids [105]. In patients with Zellweger syndrome, an inherited disorder of peroxisomal biogenesis, and a complete lack of hepatic peroxisomes, urinary excretion of dicarboxylic acids including adipic acid, suberic acid and sebacic acid was increased [121]. The association between dicarboxylic acids and the proximal tubule is further supported by the presence of sodium-dependent dicarboxylic transport systems in this region [122]. To the best of my knowledge, no reports have yet associated urinary methylsuccinate with quantitative measures of renal injury, although the strong correlations between urinary methylsuccinate and indices of glomerular injury shed further light on the potential role of impaired peroxisomal functioning in DKD progression [123].

DMT also increased urinary excretion of several PPARα-regulated nicotinamide metabolites, including 1-methylnicotinamide, nicotinamide N-oxide, and nicotinurate [124, 125]. Urinary 1-methylnicotinamide levels increase following treatment with a PPARα agonist and positively correlate with hepatic peroxisomal number [126]. Interestingly, PPARα-regulated FAO transcripts and nicotinamide metabolites were more strongly correlated with improvements in proximal tubular than glomerular injury, underscoring the importance of PPARα-regulated FAO in this region of the kidney [17, 127–129]. Using a network pharmacology approach, treatment with the PPARα agonist fenofibrate was found to be a dominant effector of PPARα-stimulated FAO in the proximal tubule following DMT [35]. The extent to which liberation of VLCFAs from adipose tissue during weight loss synergises with pharmacological PPARα agonism in the activation of renal FAO merits further investigation [130].

Among the limitations of the present study, it is noteworthy that DKD was only modelled in male rats, limiting the external validity of the findings. Sexual dimorphism with respect to extent of renal injury is recognised in preclinical models of DKD, with males typically having a more severe phenotype [131]. The extent to which our findings are replicated in female animals should be interrogated in future studies. Transcriptomic analyses were performed on whole renal cortex rather than individual cells. While *in silico* deconvolution allowed us to assign FAO genes to the proximal tubule [72, 74], dedicated single-cell transcriptomic analyses would have permitted more sensitive evaluation of cell-specific responses to DMT. Future preclinical studies evaluating the kidney single-cell or spatially resolved transcriptomic landscape are warranted to further interrogate changes in proximal tubular FAO transcript expression following weight loss plus pharmacological PPARα agonism.

Rats in the DMT group received a sham surgery, underwent dietary restriction to ~20% weight loss, and received multi-modal pharmacotherapy with five medications. The DMT interventions were conducted alongside studies of RYGB which we have previously reported on [14, 15]. In our previous preclinical studies focusing on RYGB, laparotomised rats served as a sham group and positive controls for disease progression [12–15]. To allow for potential cross-reference between RYGB and DMT results, sham surgeries were performed in the DMT groups of the ZDF and ZDSD studies. Sham surgery alone had no important effect on progression of DKD as can be inferred from combined assessment of histological, ultrastructural and molecular indices of renal injury in sham-operated rats. Together, these show clear evidence of renal disease relative to healthy controls, validating the designation of rats in this group as positive disease controls. Thus, we consider only the dietary restriction and the multi-modal pharmacotherapy components as being active in effecting the reductions in renal injury observed in rats treated with DMT.

We previously demonstrated histological evidence of partial renoprotection in ZDF rats following sham surgery and dietary restriction-induced weight loss equivalent to that achieved after RYGB, including ~33% improvement in proteinuria relative to sham-operated ZDF rats [12]. However, this was markedly inferior to reductions in proteinuria after RYGB in ZDF rats, which equated to an ~80% improvement relative to sham-operated controls [12]. Thus, while dietary restriction likely contributed to reductions in renal injury in rats treated with DMT in the present studies, it is unlikely to have been the dominant effector of these changes. It can reasonably be inferred from the transcriptomic and metabolomic profiles, and their correlations with structural and functional improvements in the kidney, that the fibrate component of DMT was the dominant factor contributing to treatment efficacy.

All five medications (fenofibrate, liraglutide, metformin, ramipril, and rosuvastatin) were provided concurrently to rats in the DMT group in an effort to maximally stimulate FAO with medications routinely used in type 2 diabetes management. As this study design makes it more difficult to discern individual medication contributions, multi-modal pharmacological treatment may be a perceived limitation of this study. However, we propose that this design is more translationally relevant to the clinical setting [34]. We employed a network pharmacology approach in which DMT vs SHAM DEGs common to both ZDF and ZDSD models were deconvoluted using curated information on medication- and PPAR isotype-responsiveness of genes obtained using IPA [71]. Fenofibrate was identified as the dominant medication effector of gene expression changes following DMT, which in turn contributed to FAO induction in the proximal tubule. A preclinical systematic review and meta-analysis assessing the impact of pharmacological PPARα activation in experimental renal injury is ongoing [132], and may help to inform the design of future preclinical and clinical studies of fibrate therapy in DKD.

On reflection, the pairing of dietary restriction with timing of medication administration may have optimised certain drug responses in rats treated with DMT, in particular the response to fenofibrate. By scheduling drug dosing to occur coincident with daily ration provision, it is possible that we paired a fasting-related circadian peak in renal PPARα expression and activity with PPARα-directed pharmacotherapy [133]. The primary aim of this study was to determine if we could non-invasively mimic the effects of bariatric surgery with a combined dietary and pharmacotherapy intervention. We cannot definitively isolate out how the components of the DMT intervention synergised to elicit renoprotection. The extent to which dietary restriction synergises with specific drug classes, particularly fibrate-based treatment, should be explored in future studies.

In summary, the impact of RYGB plus FAO-directed medications on induction of peroxisomal and mitochondrial FAO as well as reductions in renal injury was replicated in a non-invasive fashion in two Zucker rat models of DKD by combining RYGB-equivalent weight loss (~20%) through dietary restriction with fenofibrate, liraglutide, metformin, ramipril, and rosuvastatin. Fenofibrate was found to be the dominant medication effector of PPARα-regulated FAO induction in the proximal tubule, and its gene targets and related urinary metabolites were moderately-to-strongly correlated with improvements in glomerular and proximal tubular structural parameters. Thus, the combination of weight loss plus pharmacological PPARα agonism merits further investigation as a means of attenuating DKD progression.

## Supplementary Material

Supplementary Figure 1

Supplementary Figure 2

Supplementary Figure 3

Supplementary Figure 4

Supplementary Figure 5

Supplementary Figure 6

Supplementary Figure 7

Supplementary material

## Figures and Tables

**Table 1:** Changes in metabolic parameters in the ZDF and ZDSD DMT preclinical studies.^a,b^

	**Model**	**Pre-post intervention comparisons (absolute values)**								
		Healthy			SHAM			DMT		
		Pre	Post	p	Pre	Post	p	Pre	Post	p
Body weight (g)	ZDF	288.2±18.9	358.0±27.5	0.007	352.8±10.9	384.3±18.9	0.01	363.7±26.4	310.2±14.3	<0.001
	ZDSD	564.2±28.3	603.5±31.5	0.003	529.4±35.7	486.7±76.8	0.09	578.5±17.1	441.8±23.9	<0.001
Plasma glucose (mmol/L)	ZDF	5.9±0.5	5.5±1.1	0.36	15.5±5.4	24.0±1.9	0.02	15.9±2.2	7.4±1.5	<0.001
	ZDSD	5.3±0.2	7.6±2.0	0.05	13.3±6.4	22.2±7.2	0.02	10.6±4.8	6.5±1.0	0.16
	Model	Pre-post intervention comparisons (percentage delta change values)								
		Percentage delta change values					P-values for comparisons			
		Healthy		SHAM		DMT		SHAM vs Healthy		DMT vs SHAM	
Δbody weight (%)	ZDF	24.7±12.3		9.0±5.6		-14.5±4.1		0.03		<0.001	
	ZDSD	7.0±2.7		-8.3±10.5		-23.6±2.9		0.01		0.01	
Δplasma glucose (%)	ZDF	-6.9±17.4		73.3±72.6		-52.3±12.9		0.06		0.02	
	ZDSD	43.5±36.9		87.0±75.5		-26.5±38.1		0.21		0.02	
	Model	Study close comparisons								
		Study close values					P-values for comparisons			
		Healthy		SHAM		DMT		SHAM vs Healthy		DMT vs SHAM	
Cholesterol (mmol/L)^c^	ZDF	1.87±0.41		4.83±1.13		4.78±3.31		0.002		0.98	
	ZDSD	1.78±0.11		2.66±0.42		2.53±0.30		0.002		0.73	
Triglycerides (mmol/L)^c^	ZDF	1.08±0.52		5.49±0.74		2.75±0.52		<0.001		<0.001	
	ZDSD	0.85±0.19		2.37±1.43		1.00±0.47		0.04		0.047	

a DMT, dietary restriction plus medical therapy; SHAM, sham surgery (laparotomy); ZDF, Zucker Diabetic Fatty; ZDSD, Zucker Diabetic Sprague Dawley.

b Values are given as mean ± standard deviation. Statistical significance of within-group and between-group differences was derived using paired and unpaired t-tests, respectively. All t-tests were multiplicity-corrected using the Benjamini-Hochberg method [64].

c Plasma in ZDF model, serum in ZDSD model.

## Data Availability

RNA-seq fastq files are accessible through GEO (accession numbers: GSE117380 for ZDF data; GSE169085 for ZDSD data). Lists of DEGs (absolute fold-change ≥1.3, adjusted p-value <0.05) between the study groups presented are available on OSF, DOI 10.17605/OSF.IO/MUKRC. Along with sample metadata, the following urinary ^1^H-NMR data have been uploaded to OSF (DOI 10.17605/OSF.IO/MUKRC): raw spectra, PPM chemical shift vector, processed peak intensity matrices, and peak annotations. A PDF outlining spectral processing using the R package Speaq [84], as well as peak abundance by experimental group, for each annotated peak in the urinary ^1^H-NMR spectra has also been uploaded to the OSF repository.

## Clinical Perspectives

- As we previously showed that surgically-induced weight loss synergised with pharmacotherapy to reduce diabetic renal injury in association with activation of renal fatty acid oxidation (FAO), we conducted preclinical studies to determine if these beneficial effects could be replicated with a non-invasive bariatric mimetic intervention: dietary restriction plus medical therapy (DMT; fenofibrate, liraglutide, metformin, ramipril, and rosuvastatin).
- In two Zucker rat models of diabetic kidney disease (DKD), DMT resulted in ~20% weight loss, improved metabolic control, and reduced indices of glomerular and proximal tubular injury in association with activation of PPARα- regulated peroxisomal and mitochondrial FAO in the proximal tubule.
- By addressing a pathogenic deficit which is not targeted by current therapies for DKD, namely defective proximal tubular FAO, the combination of weight loss plus pharmacological PPARα agonism is a promising means of improving renal outcomes in people with type 2 diabetes which merits further investigation.

## Abbreviations

^1^H-NMR: proton nuclear magnetic resonance
*Acaa2*: acetyl-CoA acyltransferase 2
*Acox1*: acyl-CoA oxidase 1
AMPK: 5′ adenosine monophosphate-activated protein kinase. AUC area under the receiver operating characteristic curve
BMRB: Biological Magnetic Resonance Data Bank
cDNA: complementary deoxyribonucleic acid
CKD: chronic kidney disease
COSY: correlation spectroscopy
CPMG: Carr-Purcell-Meiboom-Gill
DAB: 3,3’-diaminobenzidine
DEG: differentially expressed gene
DKD: diabetic kidney disease
DMT: dietary restriction plus medical therapy
DNase: deoxyribonuclease
ECM: extracellular matrix
*Ehhadh*: enoyl-CoA hydratase and 3-hydroxyacyl CoA dehydrogenase
ELISA: enzyme-linked immunosorbent assay
ESKD: end-stage kidney disease
FAO: fatty acid oxidation
FF: fenofibrate
FLMRR: fenofibrate, liraglutide, metformin, ramipril, and rosuvastatin
FP: foot process
GBM: glomerular basement membrane
GEO: Gene Expression Omnibus
GIP: glucose-dependent insulinotropic polypeptide
GLP-1: glucagon-like peptide-1
GLP1RA: glucagon-like peptide-1 receptor agonist
H&E: haematoxylin and eosin
HSQC: heteronuclear single quantum coherence
HMDB: Human Metabolome Database
IPA: Ingenuity Pathway Analysis
IQR: interquartile range
IVDr: In Vitro Diagnostic Research
KTEA: Kidney Tubules Expression Atlas
LCFA: long-chain fatty acid. mRNA messenger ribonucleic acid
MUVR: Multivariate methods with Unbiased Variable selection in R
NMR: nuclear magnetic resonance
NOESY: nuclear Overhauser effect spectroscopy
OSF: Open Science Framework
PC: principal component
PCA: principal component analysis
Pdk4: pyruvate dehydrogenase kinase 4
PFPD: podocyte foot process diameter
PFPF: podocyte foot process frequency
PPAR: peroxisome proliferator-activated receptor
PPARα: peroxisome proliferator-activated receptor-alpha
PPARδ: peroxisome proliferator-activated receptor-delta
PPARγ: peroxisome proliferator-activated receptor-gamma
PPM: parts per million
PQN: probabilistic quotient normalisation
PT: proximal tubule. qRT-PCR quantitative real-time polymerase chain reaction
RAAS: renin-angiotensin-aldosterone system
RCT: randomised clinical trial
RF: random forest
rlog: regularized log
RNA: ribonucleic acid
RNA-seq: ribonucleic acid sequencing
RQ: relative quantification
RYGB: Roux-en-Y gastric bypass
SD: Sprague Dawley
SHAM: sham surgery (laparotomy). snRNA-seq single-nucleus RNA sequencing
SOP: standard operating procedure
SUSTAIN: Semaglutide Unabated Sustainability in Treatment of Type 2 Diabetes. TCA tricarboxylic acid
TEM: transmission electron microscopy
TOCSY: total correlation spectroscopy
TSP-d4: 3-trimethylsilyl propionic-2,2,3,3 acid sodium salt D4. uACR urinary albumin-to-creatinine ratio
uAER: urinary albumin excretion rate
VLCFA: very-long-chain fatty acid
WT-1: Wilms’ tumour-1
ZDF: Zucker Diabetic Fatty
ZDSD: Zucker Diabetic Sprague Dawley
