# Supplementary figures and images for: Dietary Restriction and Medical Therapy Drives PPARα-Regulated Improvements in Early Diabetic Kidney Disease in Male Rats
Source: Clin Sci (Lond). Author manuscript; Available in PMC 2022 Nov 15. (PMC7613831; doi:10.1042/CS20220205)

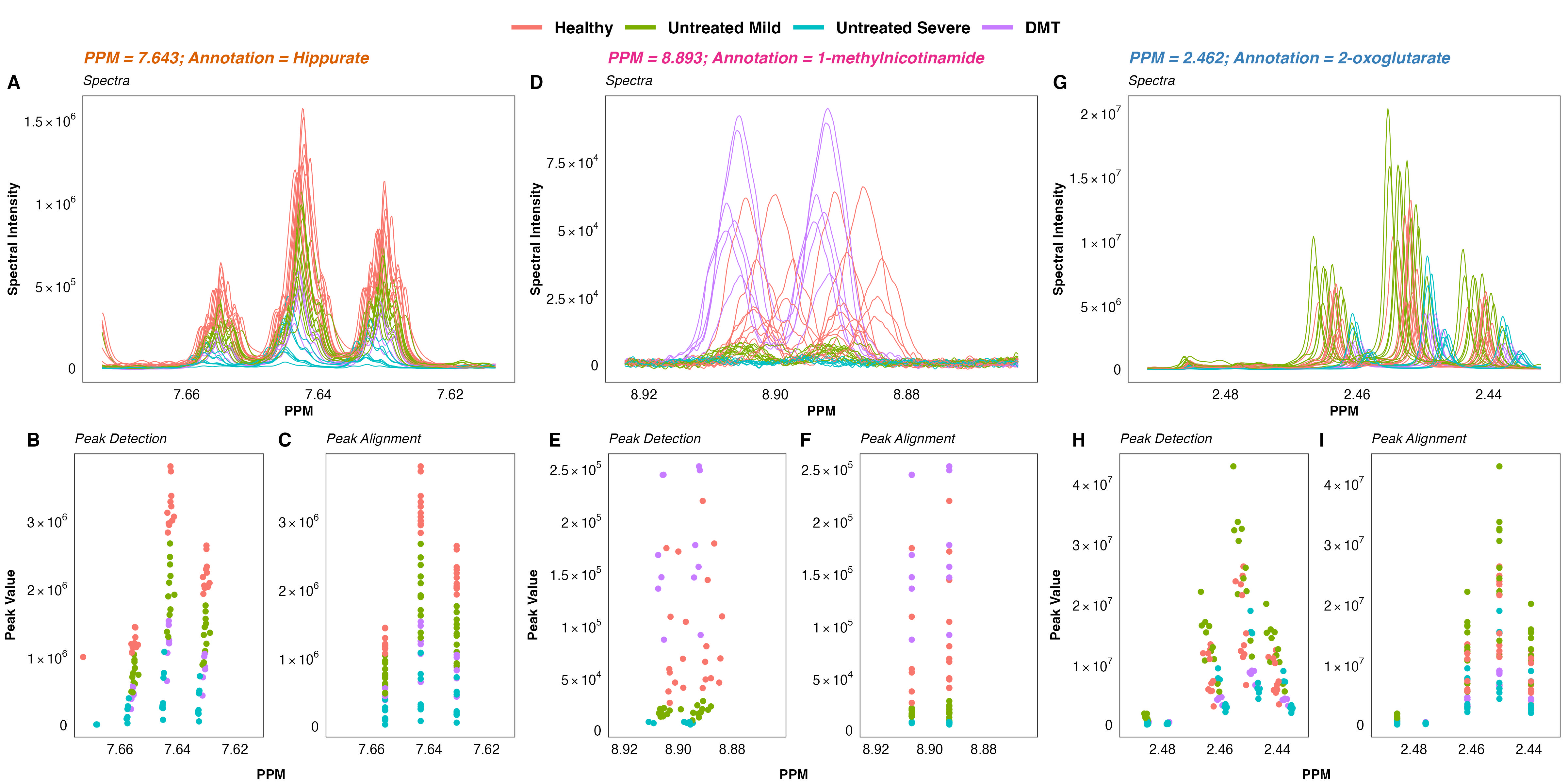

Supplement: Supplementary Figure 1 [file EMS156770-supplement-Supplementary_Figure_1.jpeg]

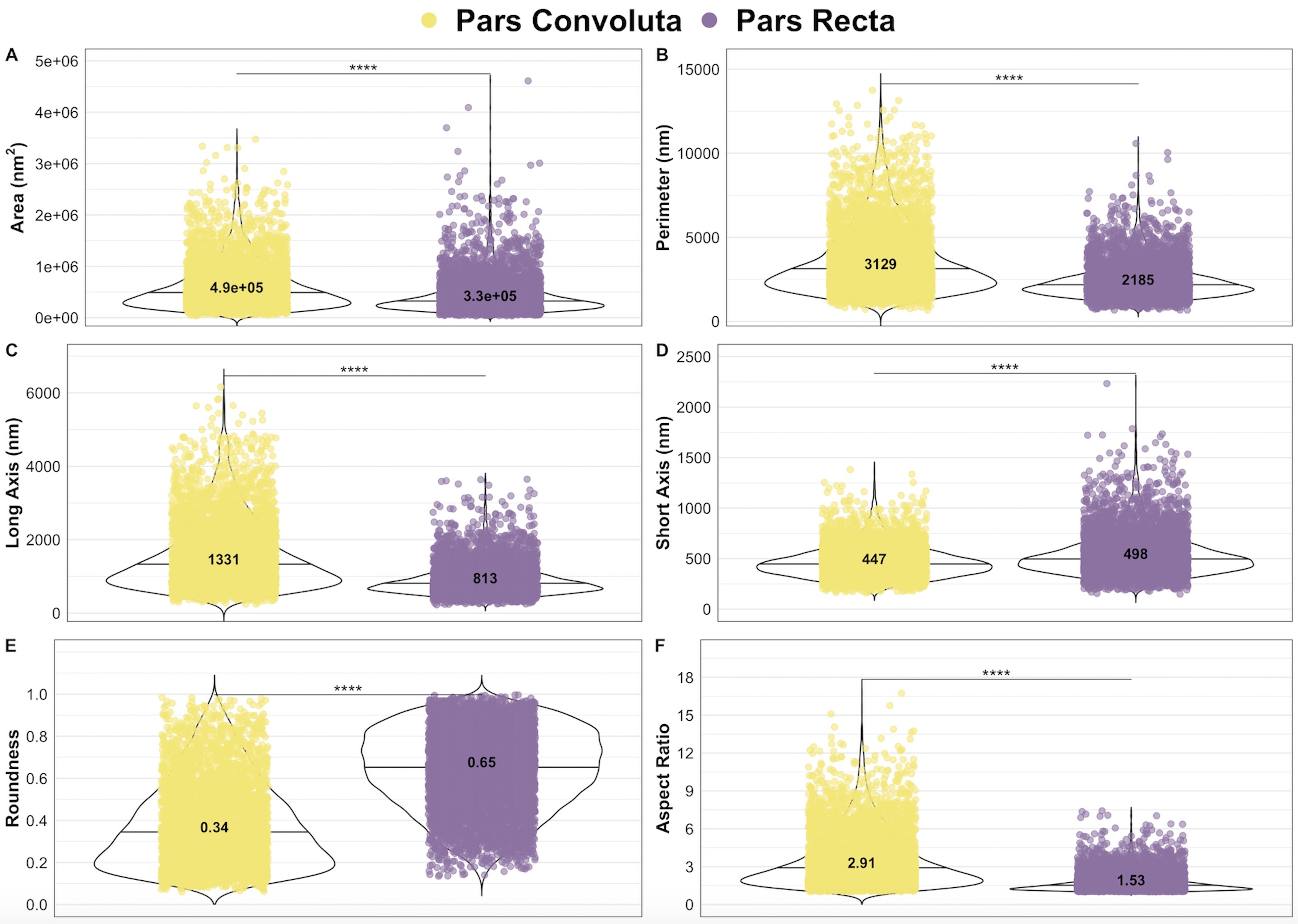

Supplement: Supplementary Figure 2 [file EMS156770-supplement-Supplementary_Figure_2.jpeg]

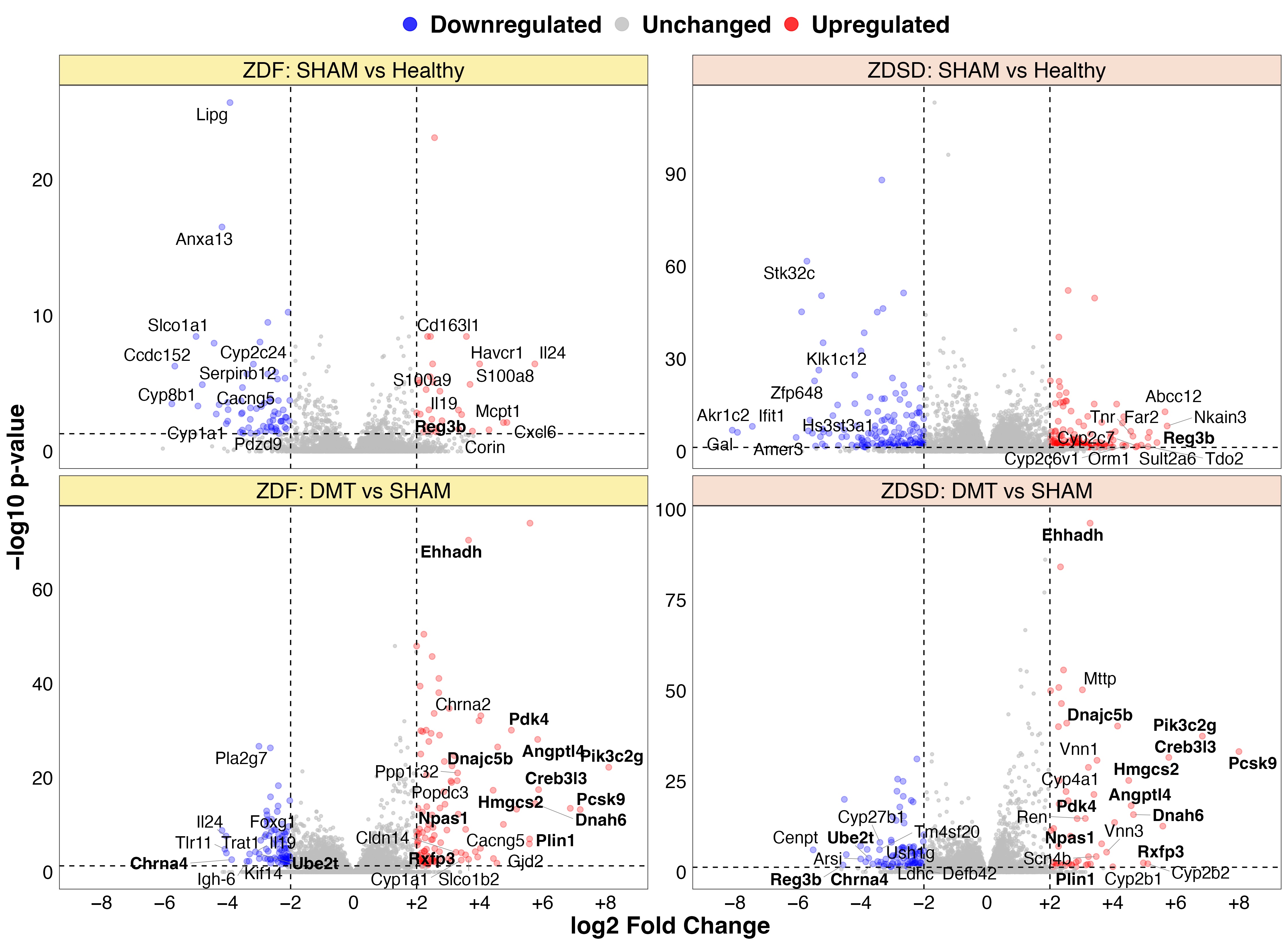

Supplement: Supplementary Figure 3 [file EMS156770-supplement-Supplementary_Figure_3.jpeg]

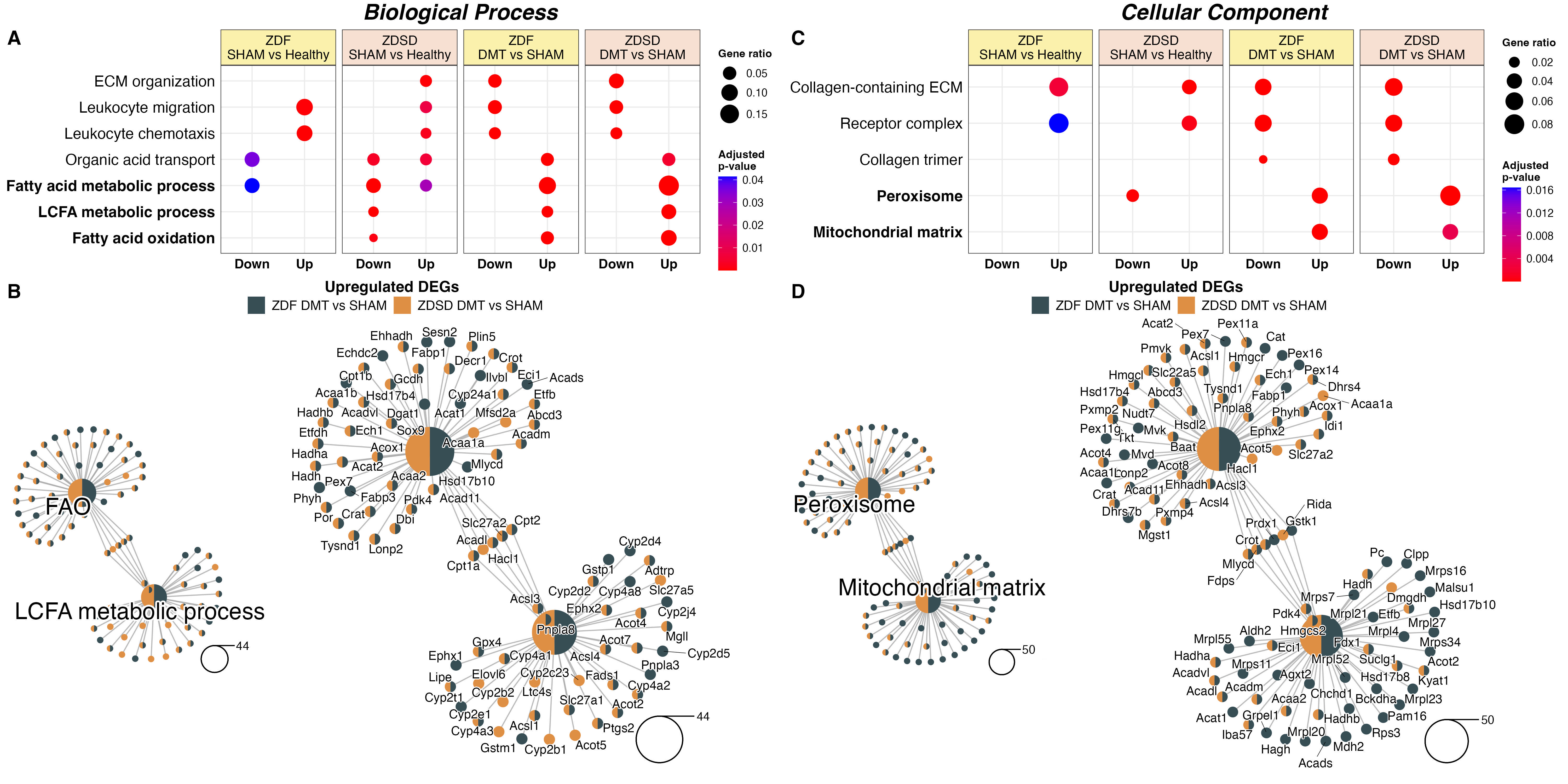

Supplement: Supplementary Figure 4 [file EMS156770-supplement-Supplementary_Figure_4.jpeg]

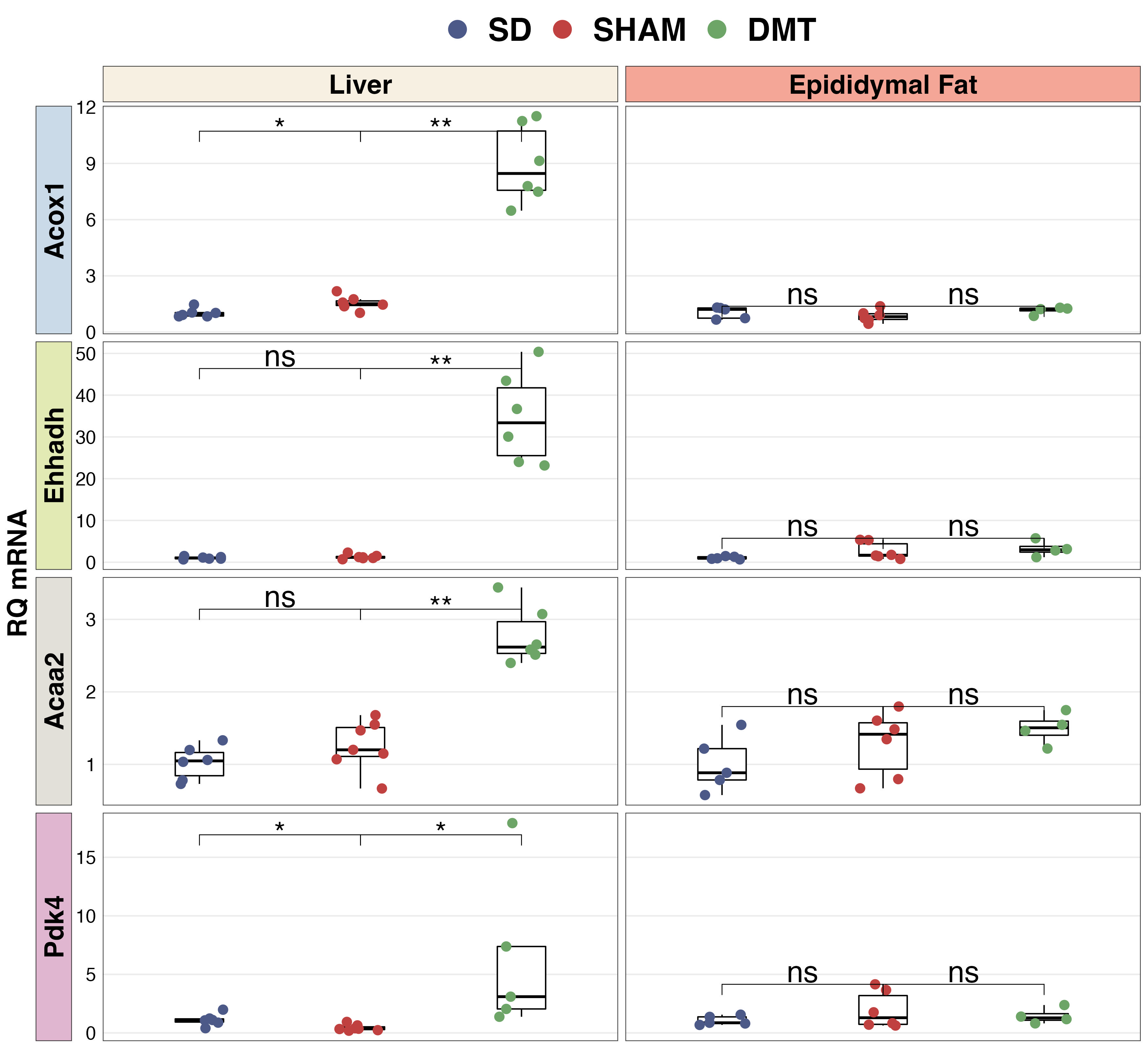

Supplement: Supplementary Figure 5 [file EMS156770-supplement-Supplementary_Figure_5.jpeg]

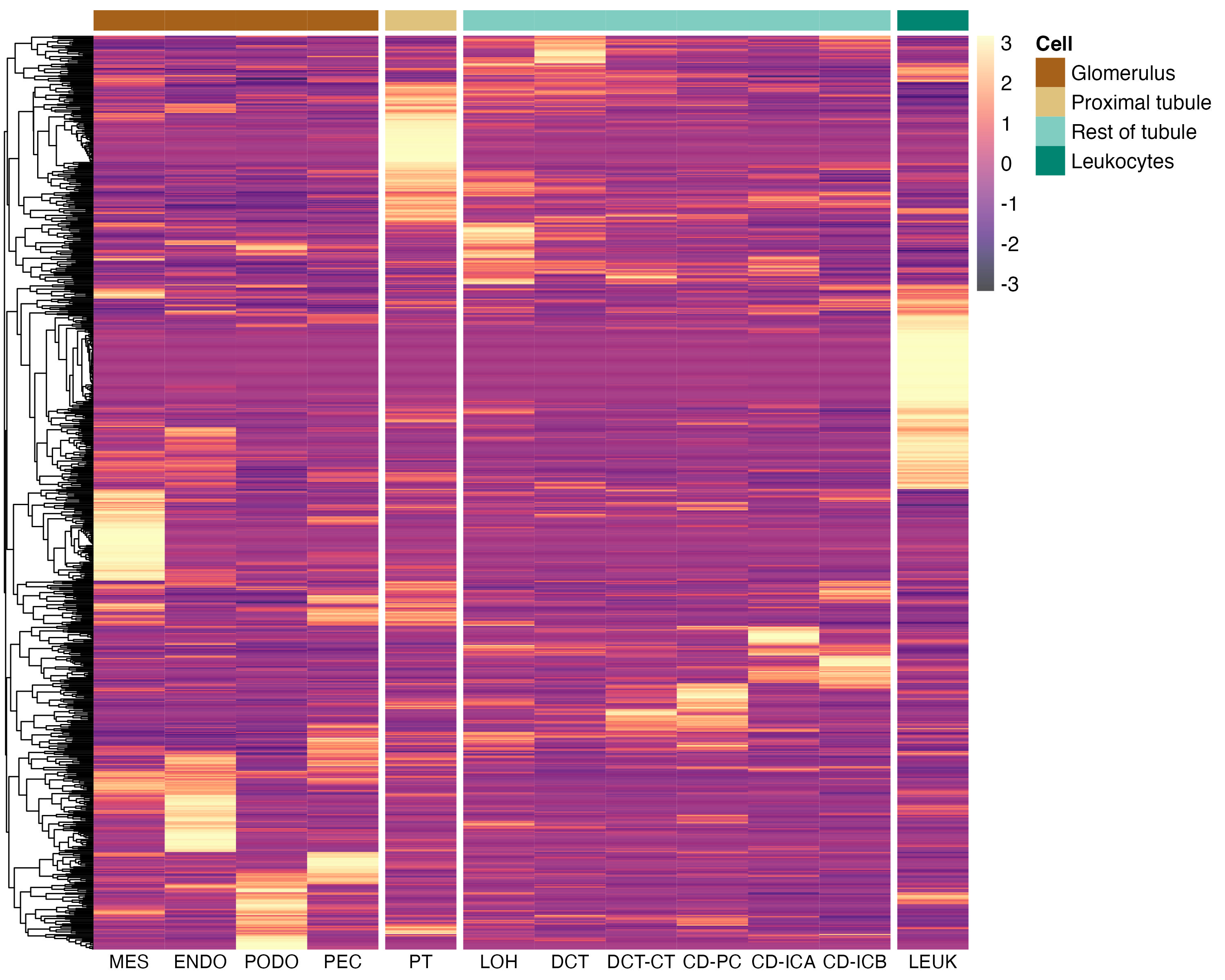

Supplement: Supplementary Figure 6 [file EMS156770-supplement-Supplementary_Figure_6.jpeg]

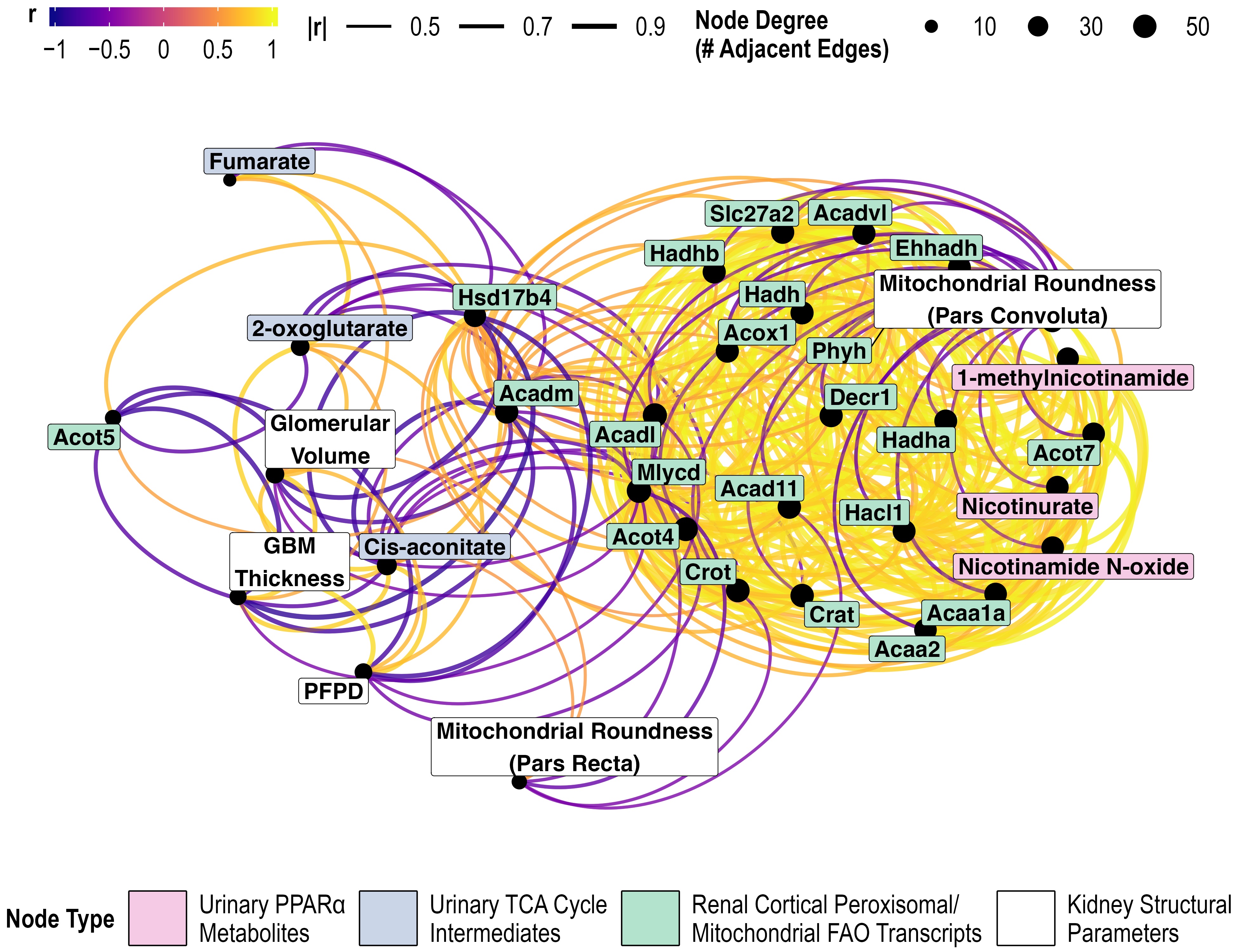

Supplement: Supplementary Figure 7 [file EMS156770-supplement-Supplementary_Figure_7.jpeg]
