# Supplementary material for: Dietary Restriction and Medical Therapy Drives PPARα-Regulated Improvements in Early Diabetic Kidney Disease in Male Rats
Source: Clin Sci (Lond). Author manuscript; Available in PMC 2022 Nov 15. (PMC7613831; doi:10.1042/CS20220205)
Supplement: Supplementary material [file EMS156770-supplement-Supplementary_material.pdf]

Supplementary Material

Dietary Restriction and Medical Therapy Drives PPARα-Regulated Improvements in Early Diabetic Kidney Disease in Male Rats

William P. Martin[1], Meera Nair[1], Yeong H.D. Chuah[1], Daniel Malmodin[2], Anders Pedersen[2], Sanna Abrahamsson[3], Michaela Hutter[1], Mahmoud Abdelaal[1], Jessie A. Elliott[1], Naomi Fearon[1], Hans Eckhardt[1], Catherine Godson[1], Eoin P. Brennan[1], Lars Fändriks[4], Carel W. le Roux[1,5], and Neil G. Docherty[1,*]

[1]Diabetes Complications Research Centre, School of Medicine, Conway Institute of Biomolecular and Biomedical Research, University College Dublin, Belfield, D04 V1W8 Dublin, Ireland
[2]Swedish NMR Centre, University of Gothenburg, 40530 Gothenburg, Sweden
[3]Bioinformatics Core Facility, Sahlgrenska Academy, University of Gothenburg, 40530 Gothenburg, Sweden
[4]Institute of Clinical Sciences, Sahlgrenska Academy, University of Gothenburg, 40530 Gothenburg, Sweden
[5]Diabetes Research Group, Ulster University, Coleraine BT52 1SA, UK
[*]Corresponding author: neil.docherty@ucd.ie

SUPPLEMENTARY FIGURES

S1 Raw spectra and spectral processing of selected urinary ^{1}H-NMR spectroscopy peaks in the ZDSD DMT preclinical study. 2
S2 Comparison of mitochondrial morphological characteristics between the pars convoluta and pars recta sections of the proximal tubule in the ZDSD study. 3
S3 Volcano plots of differentially expressed transcripts between experimental groups in the ZDF and ZDSD DMT preclinical studies. 4
S4 Biological process and cellular component gene ontology term enrichment in the ZDF and ZDSD DMT preclinical studies. 5
S5 Expression of peroxisomal and mitochondrial PPARα-responsive FAO transcripts in liver and epididymal fat tissue in the ZDSD DMT preclinical study. 6
S6 *In silico* deconvolution of the predicted cellular source of transcripts differentially expressed between DMT and SHAM rats in a human DKD snRNA-seq dataset. 7
S7 Relational network map of the correlation structure between transcripts, metabolites, and structural parameters in the renal cortical response to DMT. 8

SUPPLEMENTARY TABLES

S1 ZDF and ZDSD DMT preclinical study endpoints and statistical tests by which they were analysed. 9
S2 NMR spectroscopy characteristics of selected urinary metabolites in the ZDSD rat model. 10

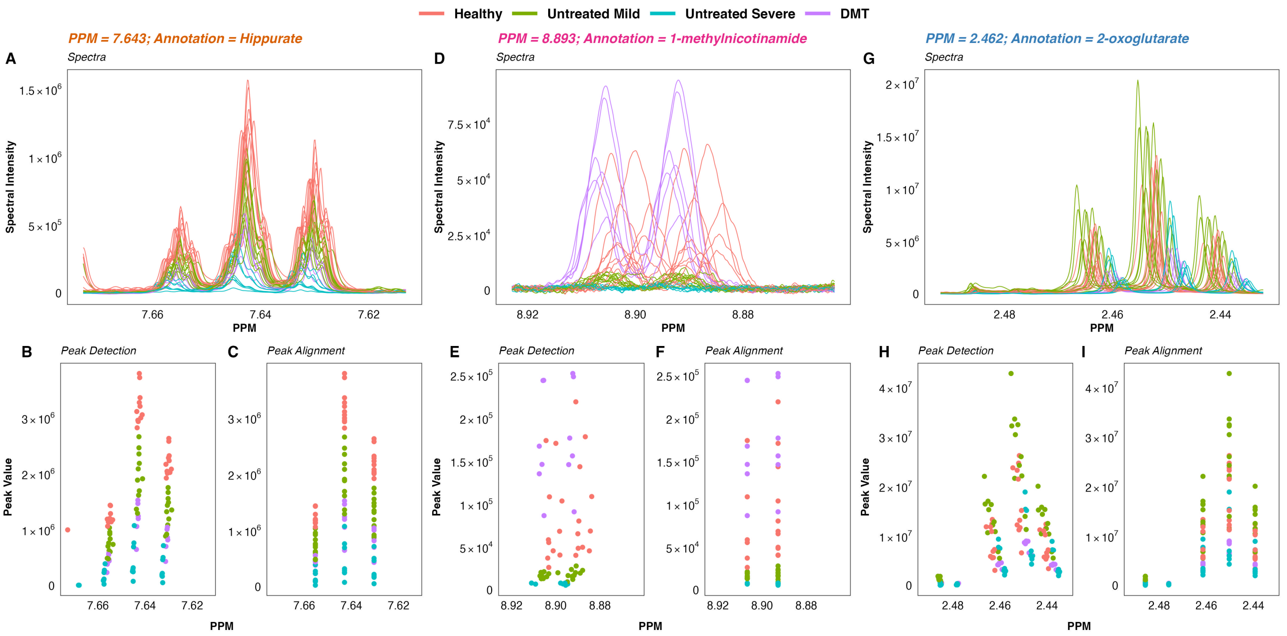

Supplementary Figure S1. Raw spectra and spectral processing of selected urinary ^{1}H-NMR spectroscopy peaks in the ZDSD DMT preclinical study.

A, D, and G: Raw ^{1}H-NMR spectra of selected peaks: hippurate, 1-methylnicotinamide, and 2-oxoglutarate. Each line corresponds to the spectrum of a single sample. B, E, and H: Detection of peaks within the spectra using a Mexican hat wavelet transformation. Each dot represents peak intensity for a single sample. C, F, and I: Alignment of peaks by grouping to account for shifts in peaks between spectra due to differences in sample environment and/or experimental conditions. Each dot represents peak intensity for a single sample. Manual inspection of the raw spectra and spectral processing in Speaq was performed as outlined here for peaks identified as important to classification of ZDSD rats by multivariate models to ensure that between-group differences in the identified peaks were not artefactual and could be reliably identified in the spectra. Healthy, n=11; untreated mild, n=11; untreated severe, n=8; DMT, n=6. ^{1}H-NMR, proton nuclear magnetic resonance spectroscopy; DMT, dietary restriction plus medical therapy; PPM, parts per million chemical shift relative to TSP-d4; PQN, probabilistic quotient normalisation; ZDSD, Zucker Diabetic Sprague Dawley.

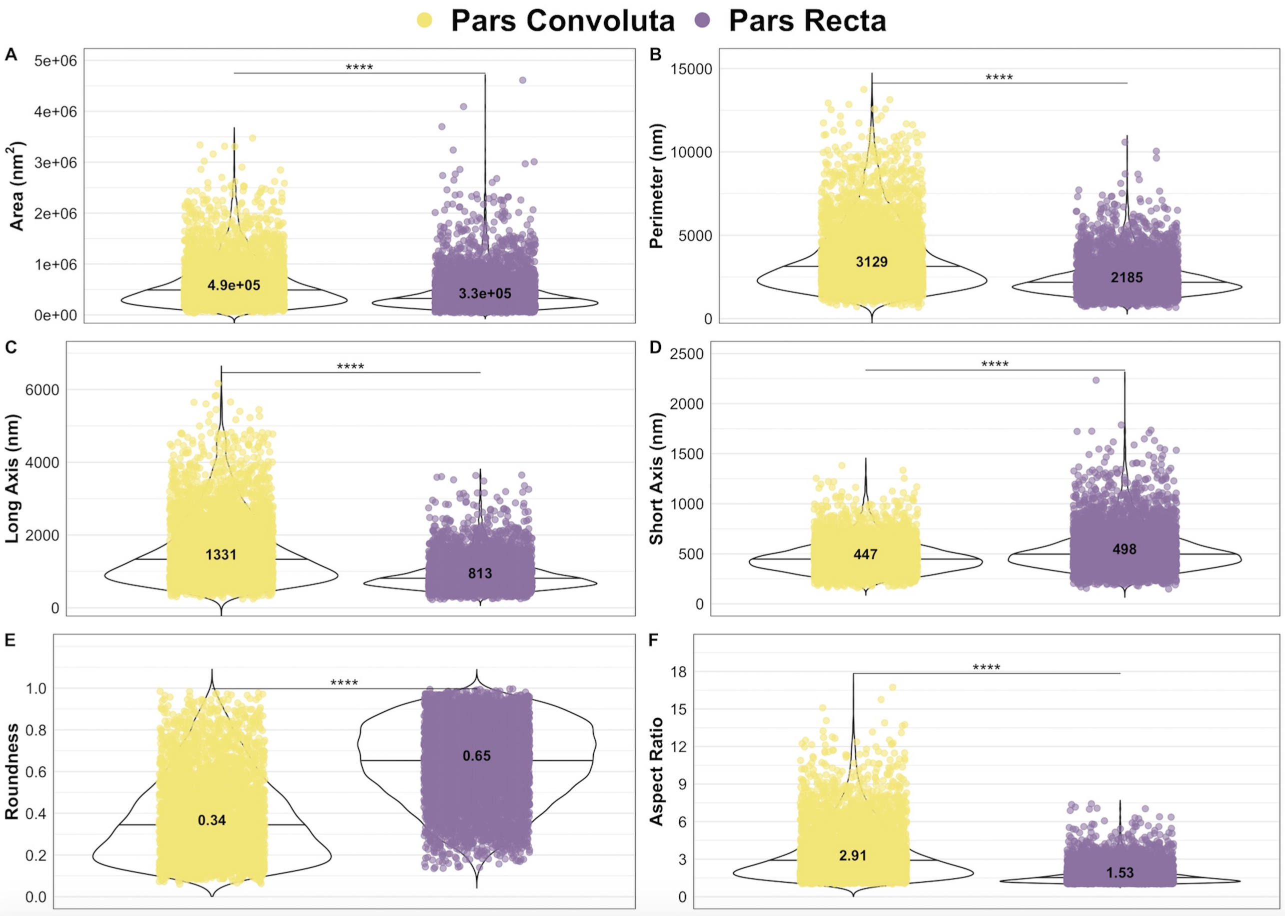

Supplementary Figure S2. Comparison of mitochondrial morphological characteristics between the pars convoluta and pars recta sections of the proximal tubule in the ZDSD study.

A-F: Values for each proximal tubular mitochondrial parameter were derived from all measurements made in both proximal tubular sections across three experimental groups: SD, SHAM, and DMT. Mitochondria were quantified in 15 non-overlapping transmission electron microscopy images captured from 3 distinct regions (5 images/region) in each of the pars convoluta and pars recta proximal tubular sections for each animal. Images from six animals were quantified in each experimental group. Data are plotted as violin plots with individual mitochondrial measurements superimposed. Median values for each proximal tubular section are identified by the horizontal black line in each violin and are also printed on each violin. Statistical significance of differences in mitochondrial characteristics between the two proximal tubular sections derived from Wilcoxon rank-sum tests is denoted as follows: ns = not significant; * = $p < 0.05$; ** = $p < 0.01$; *** = $p < 0.001$; **** = $p < 0.0001$. DMT, dietary restriction plus medical therapy; SD, Sprague Dawley.

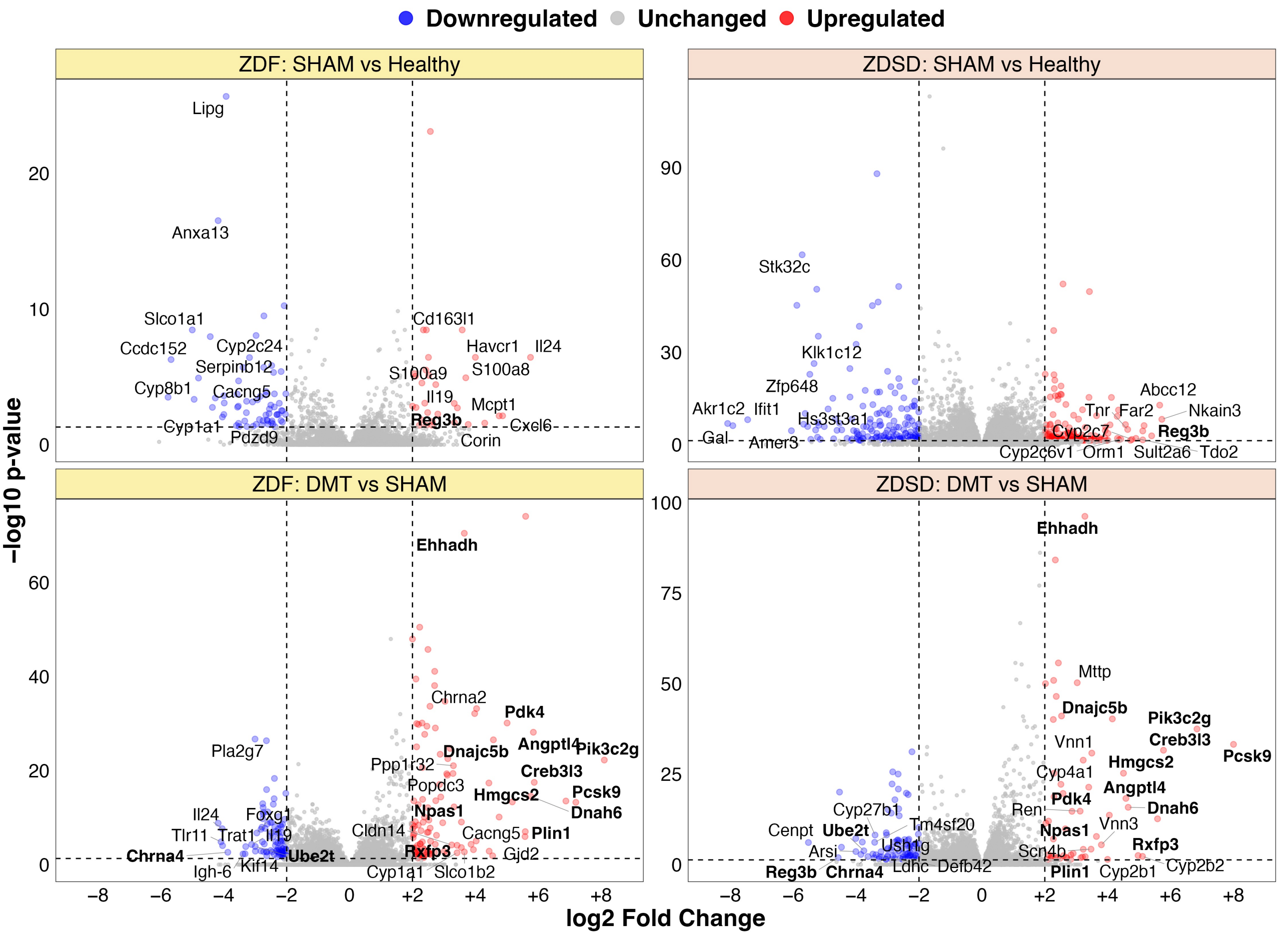

Supplementary Figure S3. Volcano plots of differentially expressed transcripts between experimental groups in the ZDF and ZDSD DMT preclinical studies.

Volcano plots of differentially expressed renal cortical transcripts between experimental groups in the ZDF and ZDSD studies [1]. Log_2 fold change is presented on the x-axis with $-log_{10}$ transformation of the multiplicity-corrected p-value for differential expression on the y-axis. Horizontal and vertical lines demarcate thresholds used to define strongly expressed transcripts: log_2 fold change values of −2 and 2, as well as a $-log_{10}$ p-value of 1.3 (corresponding to an absolute p-value of 0.05). Transcripts which were commonly changed in the ZDF and ZDSD studies are bolded. DMT, dietary restriction plus medical therapy; ZDF, Zucker Diabetic Fatty; ZDSD, Zucker Diabetic Sprague Dawley.

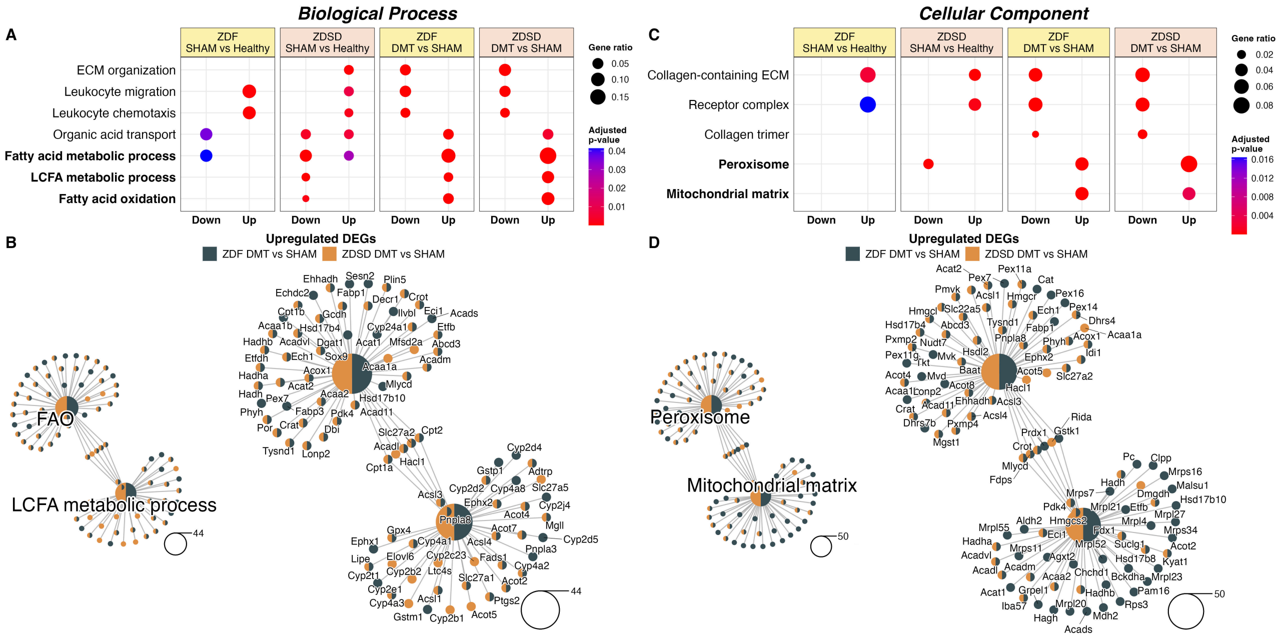

Supplementary Figure S4. Biological process and cellular component gene ontology term enrichment in the ZDF and ZDSD DMT preclinical studies.

A and C: Dotplots of biological process (A) and cellular component (C) over-representation analysis illustrating pathways commonly changed in the ZDF and ZDSD experiments, for both the SHAM vs Healthy and DMT vs SHAM comparisons [2, 3]. Gene ontology terms pertaining to fatty acid metabolism (A) and peroxisomal and mitochondrial metabolism (C) are bolded. B and D: Network plots of fatty acid metabolism biological process terms (B) and peroxisomal and mitochondrial cellular component terms (D) changed by DMT in both animal models. Smaller gene nodes, changed by DMT relative to SHAM in either or both animal models, are connected to the larger gene ontology category nodes to which they belong. For each network, gene ontology category nodes are labelled on the left and gene nodes are labelled on the right. Node size of gene ontology categories is scaled by the number of genes mapping to that category (lower right legend). Category and gene nodes are coloured according to whether they are changed in the DMT vs SHAM differential expression analysis in one or both animal models. DMT, dietary restriction plus medical therapy; ECM, extracellular matrix; FAO, fatty acid oxidation; LCFA, long-chain fatty acid; ZDF, Zucker Diabetic Fatty; ZDSD, Zucker Diabetic Sprague Dawley.

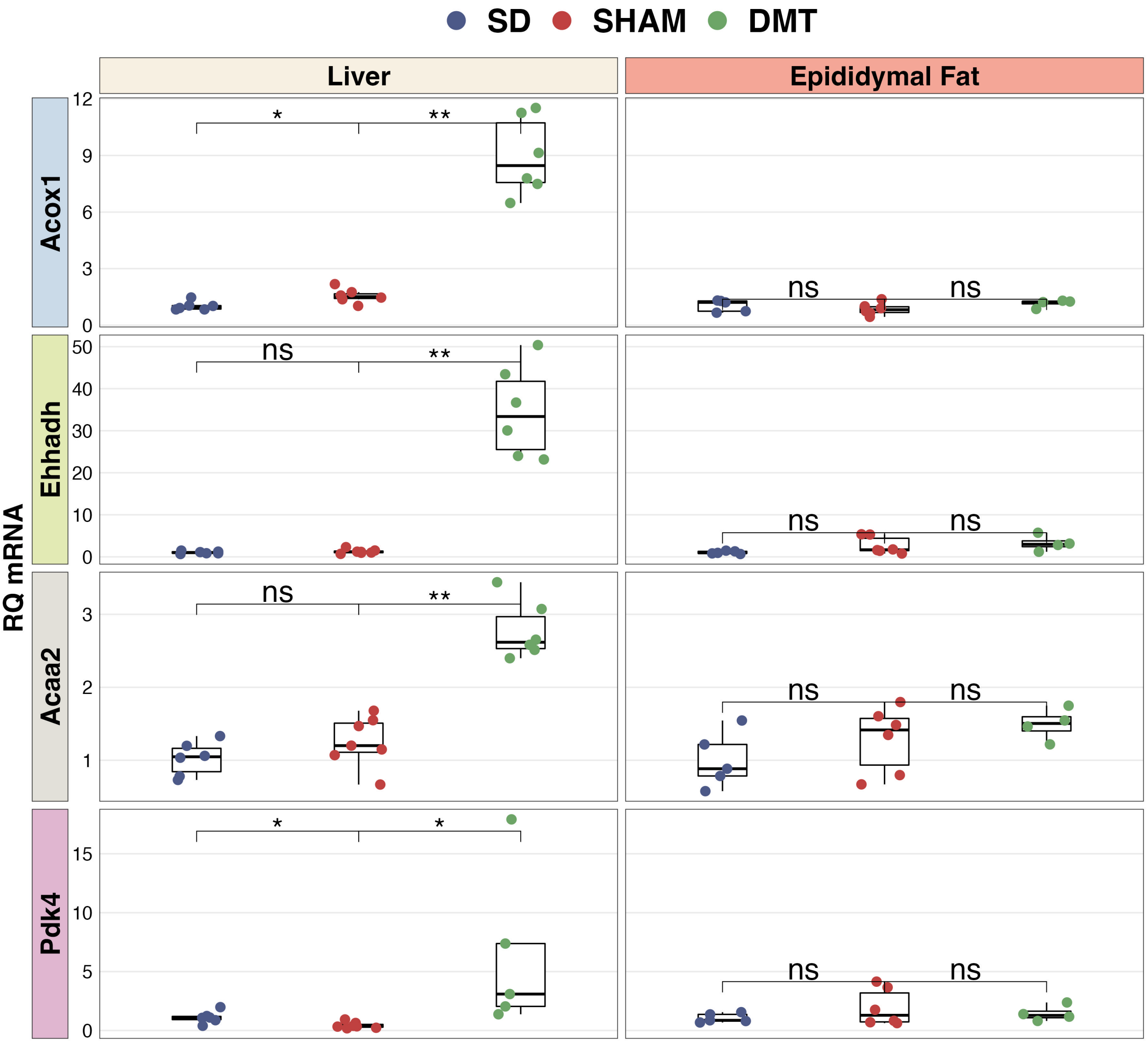

Supplementary Figure S5. Expression of peroxisomal and mitochondrial PPARα-responsive FAO transcripts in liver and epididymal fat tissue in the ZDSD DMT preclinical study.

qRT-PCR validation of expression changes in PPARα-responsive transcripts, both peroxisomal (*Acox1*, *Ehhadh*) and mitochondrial (*Acaa2*, *Pdk4*), in both liver (n=6 SD, n=7 SHAM, n=6 DMT) and epididymal fat tissue (n=5 SD, n=6 SHAM, n=4 DMT) in the ZDSD experiment. Statistical significance of between-group differences derived from multiplicity-corrected Wilcoxon rank-sum tests is denoted as follows: ns=not significant; *=p<0.05; **=p<0.01; ***=p<0.001; ****=p<0.0001. DMT, dietary restriction plus medical therapy; RQ mRNA, relative quantification of messenger ribonucleic acid; ZDSD, Zucker Diabetic Sprague Dawley.

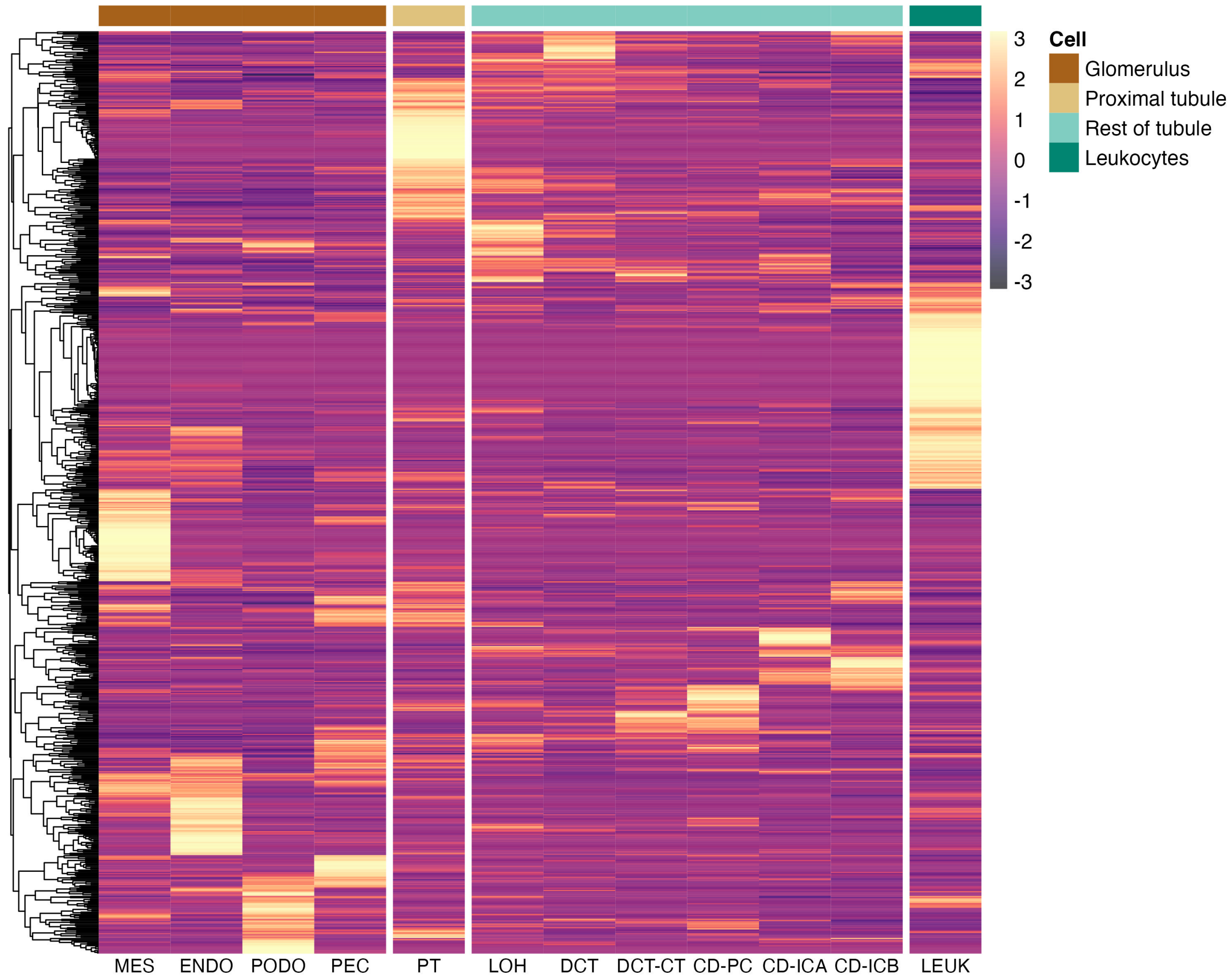

Supplementary Figure S6. *In silico* deconvolution of the predicted cellular source of transcripts differentially expressed between DMT and SHAM rats in a human DKD snRNA-seq dataset.

DMT vs SHAM DEGs commonly changed and sharing directionality between both models were intersected with a human diabetic kidney snRNA-seq dataset [4]. Transcript expression levels in the human diabetic kidney are plotted on the heatmap. Heatmap rows display DMT vs SHAM DEGs whilst each column represents 1 of 12 identified renal cell types. CD-ICA, collecting duct-intercalated cell type A; CD-ICB, collecting duct-intercalated cell type B; CD-PC, collecting duct-principal cell; DCT, distal convoluted tubule; DCT-CT, distal convoluted tubule-connecting tubule; DMT, dietary restriction plus medical therapy; ENDO, endothelial cell; LEUK, leukocytes; LOH, loop of Henle; MES, mesangial cell; PEC, parietal epithelial cell; PODO, podocyte; PT, proximal tubule; snRNA-seq, single-nucleus RNA sequencing.

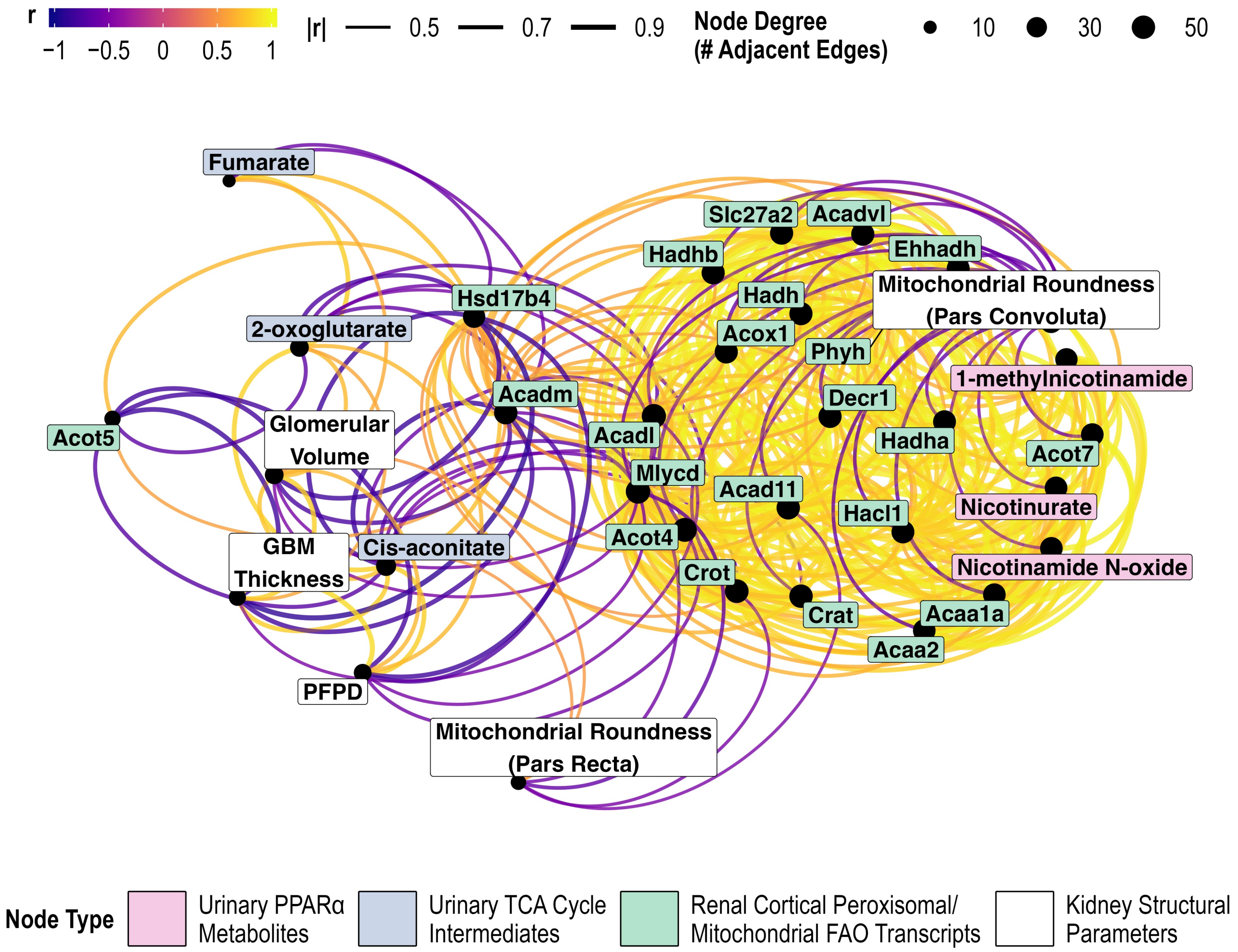

Supplementary Figure S7. Relational network map of the correlation structure between transcripts, metabolites, and structural parameters in the renal cortical response to DMT.

The network depicts Pearson correlation r values between selected renal cortical transcripts, urinary metabolites, and kidney structural parameters. Regularized log-transformed gene expression counts were used for gene-structure correlations. Selected transcripts which resulted in enrichment of peroxisomal and mitochondrial lipid metabolism pathways in ZDSD DMT rats relative to SHAM rats are plotted. PQN-normalised urinary ^{1}H-NMR peaks from samples obtained at 4 weeks after intervention were used for metabolite-structure correlations. Correlations for TCA cycle intermediates and PPARα biomarker metabolites involved in nicotinamide metabolism, many of which were differentially abundant between DMT-treated and untreated ZDSD rats, are plotted. Each node represents a variable and node labels are coloured according to variable type. Node size is scaled by the node degree, reflecting the number of edges connected to that node. Clustering of node positions is based on multidimensional scaling of absolute correlation values. Each edge represents a correlation between two variables. Edge colour indicates strength and directionality of the correlation. Edge width is scaled according to absolute correlation values. Only correlations with $|r| \geq 0.5$ are presented in the network. DMT, dietary restriction plus medical therapy; GBM, glomerular basement membrane; PFPD, podocyte foot process diameter; PPARα, peroxisome proliferator-activated receptor-alpha; PQN, probabilistic quotient normalisation; TCA, tricarboxylic acid; ZDSD, Zucker Diabetic Sprague Dawley.

Supplementary Table S1. ZDF and ZDSD DMT preclinical study endpoints and statistical tests by which they were analysed.[a,b]

Endpoint	Rat Model	Statistical Method	Location	Unit of Analysis
Within-group differences in body weight and plasma glucose from baseline to follow-up	ZDF<br>ZDSD	Paired t-test with multiple testing correction	Table 1	Per animal
Between-group differences in percentage delta change in body weight and plasma glucose from baseline to follow-up, and in circulating cholesterol and triglycerides at study close	ZDF<br>ZDSD	Unpaired t-test with multiple testing correction	Table 1	Per animal
Differences in albuminuria from baseline to follow-up in the DMT group	ZDF<br>ZDSD	Wilcoxon signed-ranked test	Figure 2A-D	Per animal
Between-group differences in glomerular histological (glomerular volume) and ultrastructural (PFPF, PFPD, and GBM thickness) parameters	ZDF<br>ZDSD	Unpaired t-test with multiple testing correction	Figure 2F,H	Per animal
Differences in mitochondrial morphology between the pars convoluta and pars recta proximal tubular sections	ZDSD	Wilcoxon rank-sum test	Supplementary Figure S2	Per individual structural measurement
Between-group differences in proximal tubular mitochondrial roundness	ZDSD	Wilcoxon rank-sum test with multiple testing correction	Figure 3C,D	Per individual structural measurement
Between-group differences in qRT-PCR data	ZDF (kidney)<br>ZDSD (kidney, liver, epididymal fat)	Wilcoxon rank-sum test with multiple testing correction	Figure 4G<br>Supplementary Figure S5	Per animal
Correlations between renal cortical transcripts, urinary metabolites, and morphometric parameters of glomerular and proximal tubular injury	ZDSD	Pearson correlations	Figure 9<br>Supplementary Figure S6	Per animal

[a] DMT, dietary restriction plus medical therapy; GBM, glomerular basement membrane; PFPD, podocyte foot process diameter; PFPF, podocyte foot process frequency; qRT-PCR, quantitative real-time polymerase chain reaction; ZDF, Zucker Diabetic Fatty; ZDSD, Zucker Diabetic Sprague Dawley.

[b] Multiple testing corrections were applied using the Benjamini-Hochberg method [5].

Supplementary Table S2. NMR spectroscopy characteristics of selected urinary metabolites in the ZDSD rat model.[a]

Metabolite(s)	PubChem Compound ID	HMDB ID	^{1}H PPM Shift	^{13}C Shift (HSQC)	TOCSY Cross Peaks	Chenomx Fit/All Metabolite Clusters
**PPARα-responsive nicotinamide metabolites**						
Nicotinurate	68499	0003269	8.943	150.24	ND	3/6
1-methylnicotinamide	457	0000699	8.893	146.16	8.959, 8.192, 9.273	4/5
Nicotinamide N-oxide	72661	0002730	8.741	ND	NE	2/6
**TCA cycle intermediates**						
2-oxoglutarate	51	0000208	2.462	33.44	3.016	2/2
Fumarate	5460307	0000134	6.532	137.92	NE	1/1
Cis-aconitate	643757	0000072	3.130	46.40	5.740	2/2
**Metabolites reflective of kidney clearance**						
Hippurate	464	0000714	7.643	134.75	7.561, 7.844	4/5
3-indoxyl sulfate	10258	0000682	7.516	114.81	7.701, 7.279, 7.199	2/6
Methylsuccinate	10349	0001844	1.088	20.03	2.15, 2.53, 2.63	1/4

[a] HMDB, Human Metabolome Database; HSQC, heteronuclear single quantum coherence spectroscopy; ND, not determined (signal too weak); NE, no cross peak expected; NMR, nuclear magnetic resonance; PPARα, peroxisome proliferator-activated receptor-alpha; PPM, parts per million chemical shift relative to TSP-d4; TCA, tricarboxylic acid; TOCSY, total correlation spectroscopy.

SUPPLEMENTARY REFERENCES

1. Love MI, Huber W, and Anders S. Moderated estimation of fold change and dispersion for RNA-seq data with DESeq2. *Genome Biology* (2014); 15 (12): 550. DOI: 10.1186/s13059-014-0550-8.
2. Yu G, Wang LG, Han Y, and He QY. clusterProfiler: an R package for comparing biological themes among gene clusters. *Omics: a journal of integrative biology* (2012); 16 (5): 284–7. DOI: 10.1089/omi.2011.0118.
3. Wu T, Hu E, Xu S, Chen M, Guo P, Dai Z, et al. clusterProfiler 4.0: A universal enrichment tool for interpreting omics data. *The Innovation* (2021); 2 (3): 100141. DOI: 10.1016/j.xinn.2021.100141.
4. Wilson PC, Wu H, Kirita Y, Uchimura K, Ledru N, Rennke HG, et al. The single-cell transcriptomic landscape of early human diabetic nephropathy. *Proc Natl Acad Sci U S A* (2019); 116 (39): 19619–25. DOI: 10.1073/pnas.1908706116.
5. Benjamini Y and Hochberg Y. Controlling the false discovery rate: a practical and powerful approach to multiple testing. *Journal of the Royal statistical society: series B (Methodological)* (1995); 57 (1): 289–300.
